# Peduncle Necking in *Rosa hybrida* Induces Stress-Related Transcription Factors, Upregulates Galactose Metabolism, and Downregulates Phenylpropanoid Biosynthesis Genes

**DOI:** 10.3389/fpls.2022.874590

**Published:** 2022-04-18

**Authors:** Bianca Lear, Matthew Casey, Anthony D. Stead, Hilary Joan Rogers

**Affiliations:** ^1^School of Biological Sciences, Royal Holloway University of London, Egham, United Kingdom; ^2^School of Biosciences, Cardiff University, Cardiff, United Kingdom

**Keywords:** floral senescence, peduncle, *Rosa hybrida*, stress biology, transcriptome

## Abstract

Roses are highly valued as cut flowers worldwide but have limited vase life. Peduncle bending “bent neck” or “necking” is a major cause of reduced vase life, especially in some cultivars. Necking is thought to be caused by either an air embolism or accumulation of microorganisms at or within the stem end, blocking the xylem vessels and preventing water uptake. However, the underlying mechanisms of necking are poorly understood. Here, RNAseq analysis was applied to compare gene expression across three stages of peduncle necking (straight, <90°, and >90°), in the necking-susceptible *Rosa hybrida* cultivar H30. Most gene expression change was later in bending and there was, overall, more downregulation than upregulation of gene expression during necking. Photosynthetic, starch, and lignin biosynthesis genes were all downregulated, while genes associated with galactose metabolism, producing raffinose and trehalose that are both related to osmoprotection, were upregulated. Genes associated with starch breakdown, autophagy, and senescence were also upregulated, as were most of the NAC and WRKY transcription factors, involved in stress and senescence regulation. Microscopy showed a cellular collapse in the peduncle. These data support a possible mechanism, whereby a reduction in water transport leads to a cellular collapse in the peduncle, accompanied by upregulation of senescence and drought responses.

## Introduction

Roses (*Rosa hybrida*) are important cut flowers, valued for their wide range of colors, shapes, and in some cases, fragrance. However, a flower's life span is limited (Fanourakis et al., 2013). In most flowers, senescence is driven by the need for the plant to limit resources and is often terminated by pollination. This can be associated with a burst of the growth regulator, ethylene (Ma et al., 2018). However, rose deterioration, especially in the vase, can also be affected by other factors. Rose vase life is typically terminated by wilting, browning, or blueing of the petals, petal abscission, visible infection (e.g., by *Botrytis cinerea*), or bending of the peduncle, also known as “necking” (van Meeteren et al., 2015). Necking is thought to be caused by vascular occlusions of the xylem, preventing water uptake (Bleeksma and van Doorn, 2003), and is a primary and early cause of post-harvest loss in cut roses. It is, therefore, a major issue for the cut flower industry. The vascular occlusions causing necking can be caused solely by air embolisms, introduced through cutting the stem, or also by microbial cells at the stem end, or in the vase water, causing a blockage of the xylem (Bleeksma and van Doorn, 2003). Although the association between microbial contamination and necking is widely accepted, there is still considerable variation in occurrence amongst rose cultivars and amongst individual stems (Bleeksma and van Doorn, 2003), even when held in the same vase water. This indicates that other factors may also be important.

Necking occurs at the peduncle, located between the receptacle, and the uppermost internode of an inflorescence stem. This is a weak point in the stem as there is a change in the morphology of the primary xylem at the transition point between the stem and the peduncle, causing a point of reduced hydraulic conductance (Darlington and Dixon, 1991). This, in turn, is thought to induce cavitation in the peduncle and limit water to the flower head in times of water stress to maintain water supply to the main axis of the plant (Darlington and Dixon, 1991). Moreover, due to a lack of secondary growth (including secondary xylem and phloem), the peduncle is less lignified than the rest of the stem and is, therefore, also a point of reduced mechanical strength. The water balance, as well as the development of vascular bundles, the thickness of the epidermis, and cell wall structure and composition, all contribute to stem strength in *Rosa hybrida* cut flowers (Chabbert et al., 1993; Matsushima et al., 2010). Disruptions to water balance during vase life and loss of turgor pressure in the peduncle result in a reliance on stem architecture to remain upright (Chabbert et al., 1993). Cultivar variation in susceptibility to necking is thought to be related to differences in mechanical stem strength, with increased mechanical strength shown to increase resistance to peduncle necking (Zamski et al., 1991).

The physiological process of reduced water uptake and bending of the peduncle, resulting in necking, is likely to affect the biochemistry and, hence, the peduncle tissue's gene expression. In *Gerbera jamesonii* (gerbera), which also suffers from stem bending, bending was associated with stem water loss and differences in lignin levels (Perik et al., 2014). Transcriptomic analysis in gerbera stems also indicated the involvement of water stress, with enrichment of pathways related to phenylalanine metabolism, starch and sucrose metabolism, and plant hormone signal transduction (Ge et al., 2019). However, stem bending in gerbera is likely to be substantially different from rose necking due to the very different stem anatomy and the less clear involvement of microbial causes (de Witte et al., 2014). In *Populus trichocarpa*, a transcriptome of xylem parenchyma cells following stem embolism formation showed enrichment of genes related to aquaporins, ion transporters, and carbohydrate metabolism (Secchi et al., 2011). Expression of aquaporin, sugar metabolism and flavonoid synthesis genes was also perturbed in grapevine petioles during rehydration after water stress (Perrone et al., 2012). Aquaporins are small transmembrane proteins important for the transport of water and small neutral solutes across biological membranes (Maurel et al., 2008). They can be divided into subfamilies including plasma membrane intrinsic proteins (PIPs), tonoplast intrinsic proteins (TIPs), Nodulin-like intrinsic proteins (NIPs), and the less well-characterized small basic intrinsic proteins (SIPs) (Maurel et al., 2008). The PIPs make up the largest subfamily and are known to be highly responsive to environmental stimuli, including drought stress and microbial infection, to help control and maintain water homeostasis (Maurel et al., 2008). Changes in the expression of transcription factors were also noted in the *Populus* study with strong responses from *AP2-EREBP, bZIP, HSF, MYB, MYB-related*, and *WRKY* family genes (Secchi et al., 2011). Expression of *NAC, MYB, WRKY*, and *AP2/ERF* family transcription factors including *DREB/CBF* genes involved in ABA-independent expression during drought was also altered during stem bending in gerbera (Ge et al., 2019).

To better understand the processes involved in rose peduncle necking, susceptible and non-susceptible cultivars were identified. Transcriptomic analysis was carried out on three physiological stages of the necking-susceptible cv. H30 and verified by qPCR on peduncles in which necking was induced. We show changes in the expression of transcription factors, aquaporin genes, stress, and metabolic pathways. These changes are consistent with a water-deficit stress response resulting in the activation of stress and senescence-related pathways.

## Materials and Methods

### Plant Material and Vase Conditions

*Rosa hybrida* cultivars, Akito, Fuchsiana, Furiosa, H30, Topsun, and Tropical Amazon, were grown on commercial flower farms in Naivasha, Kenya, and transported dry to the UK *via* air freight. Stems were collected within a day of arrival, transported dry to the laboratory, and rehydrated at 5°C overnight in tap water before being placed in vases. Stem ends were cut upon arrival to the laboratory and prior to being placed in vases, cut to a final stem length of 47 cm. Any foliage showing visible damage or on the lower 20 cm of the stem was removed prior to being placed in vases. All flower stems were kept at 21°C for the duration of vase life, with a 12 h light cycle of 15–20 μmol in m^−2^ and s^−1^, from cool white fluorescent tubes. Stems were placed in either 100 ml tap water, 2% sucrose (w/v), or commercial rose flower food (Chrysal) at the manufacturers' recommended concentration to replicate consumer conditions, in bunches, or individually in clear glass vases. Vases were arranged on benches in a completely randomized design and re-ordered every few days to reduce the effect of placement within the vase life room.

### Vase Life

The vase-life was determined as the time from stems being placed in the vase (tap water 1 L in bunches of 8 stems with three replicate bunches) until a termination factor was reached. Flower quality was assessed daily, with the day of termination and terminating factor recorded for each stem. Termination factors included the following: bending of the peduncle (necking), visible color change (blueing or browning) of the petals, and wilting.

### Relative Fresh Weight of Each Necking Stage

**Figure 1:**
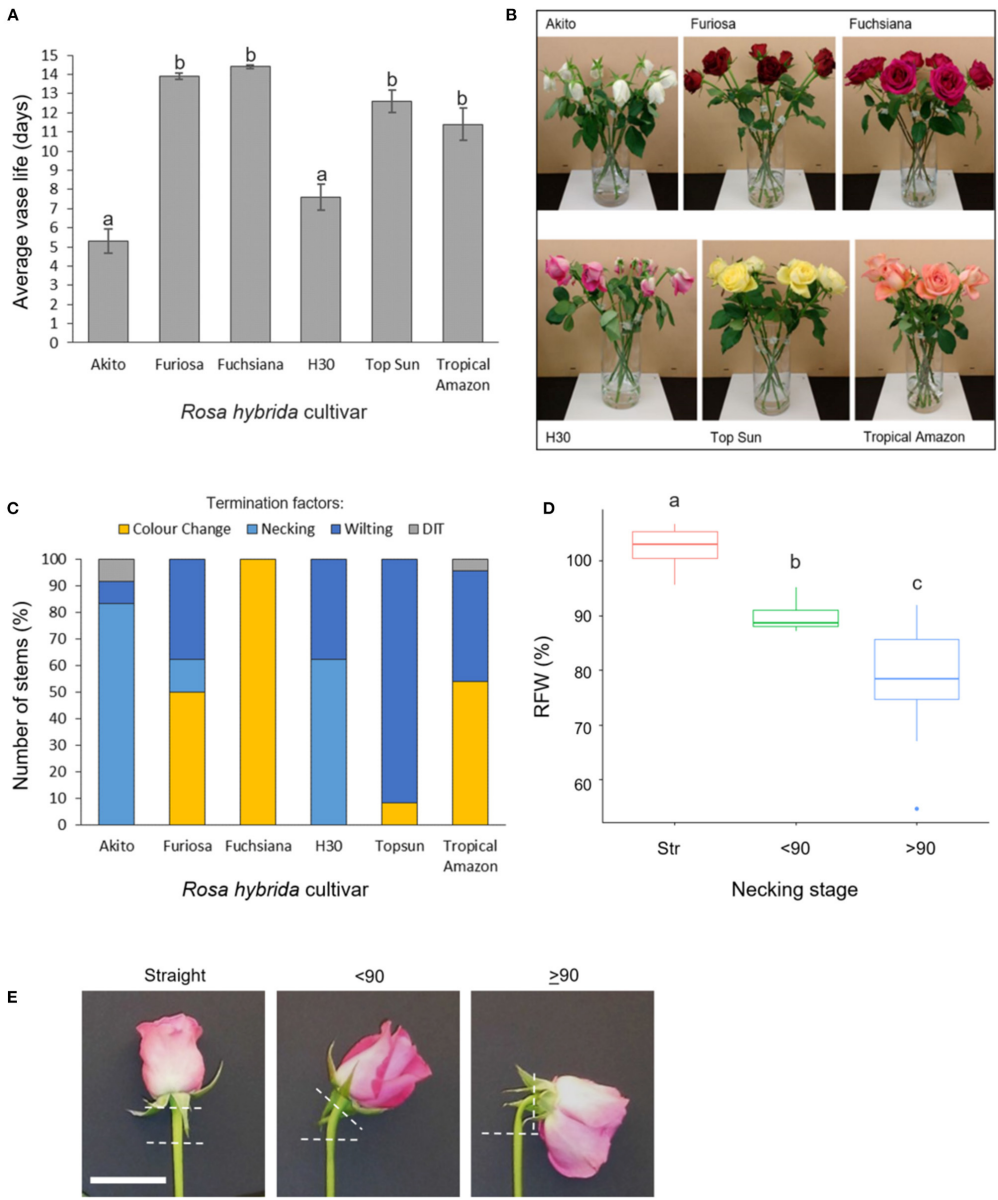
Vase life, incidence, and effects of necking in six *Rosa hybrida* cultivars **(A)** vase life (mean ± SE; *n* = 24); **(B)** images representative of termination factors seen at day 9 of vase life; **(C)** termination factors of vase life for each cultivar; color change includes both blueing and browning of petals; DIT, damaged in transit (*n* = 24). **(D)** relative flower head fresh weight of “H3O” and “Fuschiana” rose stems at three necking stages (mean ± SE ; *n* = 11–21). Different letters indicate significant differences (*p* < 0.05), based on ANOVA with a Tukey *post hoc* test. **(E)** images of the three necking stages in “H3O” (scale bar = 5 cm).

Individual stems of “H30” and “Fuchsiana” were held in 2% sucrose with the addition of *Pseudomonas fluorescens* (5 × 10^4^ cfu per mL) to induce necking. Stems were weighed to the nearest mg just prior to being placed in vases. Stems at each physiological necking stage (straight, <90°, and >90°; Figure 1E) were harvested and weighed as necking occurred. The percentage relative fresh weight (RFW %) for each necking stage was calculated using Equation 1, where W_t_ was the weight of the stem (g) at harvest and W_t = 0_ was the weight of the stem (g) at the start of vase life.

*Equation 1:*


(1) $$\begin{array}{r} RFW\;\left(\%\right)=\;\left(\frac{W_{t}}{W_{t=0}}\right)*100 \end{array}$$

### Microscopy

Necking was induced in ‘H30', as above, using *Pseudomonas fluorescens*. Peduncle sections (1 cm) for straight, <90°, and >90° physiological necking stages were excised using a sterile razor blade and placed immediately in a standard formaldehyde alcohol acetic acid (FAA) fixative solution (Ruzin, 1999). Samples were fixed overnight at 5°C and stored at 5°C until used.

For light microscopy, peduncle samples were fixed in FAA, rinsed and dehydrated in graded ethanol at concentrations from 30% to absolute ethanol (1 h for each). After overnight in absolute ethanol, they were transferred to acetonitrile for 1 h, followed by infiltration with a 50:50 acetonitrile/ Spurr resin mixture for 24 h. Peduncles were then infiltrated with 100% Spurr resin and polymerised at 60°C. Transverse sections (500 nm) were cut using a microtome and stained with toluidine blue. Slides were scanned using an Olympus BX51 microscope, with an Olympus CC12 color microscope camera and an Olympus dotSlide automated scanner using a 40 × objective.

For scanning electron microscopy, peduncle samples were cut into 1-mm transverse sections and mounted uncoated onto specimen stubs. A FEI Quanta 250 scanning electron microscope was used (Biomedical imaging unit, Southampton), with 10 kV accelerating voltage, 8.7 mm working distance (WD), and low vacuum mode 60 Pa chamber pressure.

### RNA Extraction and Sequencing

Bunches of “H30” stems were held in tap water with rose flower food (Chrysal) to replicate consumer conditions. As previously described (Lear et al., 2019), for each stage of necking (straight, <90°, and >90°) 2-cm stem sections were excised using a sterile razor blade and flash-frozen in liquid nitrogen. Necked and control (straight-stemmed) samples were taken as the necking stages appeared, and were stored at −80°C.

For each of the necking stages, three peduncle sections were ground to produce one pooled sample with three pooled samples per stage. Total RNA was extracted from each sample as previously described (Moazzam Jazi et al., 2015), and adapted for use with 1.5 mL microcentrifuge tubes. Genomic DNA (gDNA) contamination was removed from the RNA samples using the RapidOut DNA removal kit (Thermo Scientific). Samples were quality tested using a Qubit fluorometer and then sequenced using an Illumina NovaSeq 5000 to produce paired-end reads for each sample. One set of replicate samples was collected and sequenced in 2014 with a mean sequencing depth of 62.5 million, and the other two in 2016 with a depth of 25.3 million reads.

### Alignment to Reference Genome and Differential Expression Analysis

The Galaxy web platform (usegalaxy.org) was used to process and analyse the raw sequencing data (Afgan et al., 2016). Raw reads were filtered and trimmed using Trim Galore! v0.4.3.1 with a phred quality score cut off of 20 (Krueger, 2017). The reads were then aligned to the *Rosa chinensis* Old Blush genome v2 using HISAT v2.1.0 (Kim et al., 2015; Raymond et al., 2018).

Read counts were produced per exon using DEXSeq count v1.20.1 (Anders et al., 2012), with a minimum alignment quality threshold of 10. Differential expression analysis was completed using DESeq2 v2.11.40.1 (Love et al., 2014). Two factors were considered in the statistical model, necking stage and sequencing year, with the necking stage used as the primary factor.

### Gene Annotation

Differentially expressed genes were functionally annotated with gene ontology (GO) terms using the InterProScan annotation files for the *Rosa chinensis* genome (Jung et al., 2014). The GO terms were then grouped into broader Plant GO slim biological process groups for visual analysis. Homologous *Arabidopsis thaliana* (TAIR10) identifiers were used in enrichment analysis with AgriGO v2.0 and STRING (Tian et al., 2017; Szklarczyk et al., 2018), as identified for the *Rosa chinensis* genome using Blastp, with an e-value cut off of 1e^−6^ (Jung et al., 2014). For expression analysis of senescence-associated genes, all genes associated with the term “senescence” for *Arabidopsis thaliana* were downloaded from uniport and were analyzed using STRING. The expression of transcription factors was analyzed using the Plant transcription factor database v5.0 for *Rosa chinensis* (Jin et al., 2016).

### qPCR

Necking was induced in “H30” using *Pseudomonas fluorescens* as described above, and peduncle samples for each physiological necking stage (straight, <90°, and >90°) were collected as necking occurred. The gDNA removal and complementary DNA (cDNA) synthesis, using oligo-dT and random primers, were performed with a Quantitect Reverse Transcription Kit (Qiagen). The cDNA was 1/10 diluted with RNase-free water, to a working concentration of 5 ng/μL (assuming equal efficiency of reverse transcription) and stored at −20°C. Reactions (20 μL) were run using a Rotor-Gene Q thermocycler (Qiagen, Model: 5-Plex) with 1× Rotor-gene SYBR Green PCR master mix (Qiagen), 0.5 μM forward primer, 0.5 μM reverse primer, RNase-free water, and 10 ng cDNA (2 μl of 1/10 diluted template cDNA). All primers are listed in Supplementary Table 1. A two-step cycling program was used with an initial DNA polymerase activation at 95°C for 5 m, followed by 40 cycles of denaturation at 95°C for 5 s, and combined annealing and extension at 60°C for 10 s. All qPCR reactions were run with two technical replicates and four biological replicates for each stage. The Ct values were recorded at a 0.2 threshold. Expression levels were calculated (Pfaffl, 2001) relative to *RhGACPC2*, with the necking stage “Straight” as the control.

## Results

### Necking Is Cultivar-Dependent and Affects Both Flower Fresh Weight and Anatomy

In a comparison across six commercial rose cultivars, mean vase life varied significantly between 5 and 14 days (Figure 1A). The Cv.s H30 and Akito were found to have significantly shorter vase lives than all the other cultivars tested, with mean vase lives of 7.6 and 5.3 days respectively (*p* < 0.05) (Figure 1A). When the cause of vase life termination was assessed, all of the “H30” and “Akito” stems were terminated due to water stress-associated factors, namely, necking and wilting (Figures 1B,C). In contrast, none of the cv. Fuchsiana, Topsun, or Tropical Amazon stems were terminated due to necking during the observed 14-day vase life period. The majority of cv. Topsun stems were also terminated due to water stress-associated factors. However, in this case, only petal wilting was seen, while the “Fuchsiana” stems were all terminated due to a change in petal color, including both blueing and browning (Figure 1C). In no cultivar were 100% of stems terminated by necking.

To investigate the effects of necking in more detail, the addition of *Pseudomonas fluorescens* cultures to the vase water was tested to induce necking in both “H30” and “Fuchsiana” flower stems. Necking was consistently induced by the *P. fluorescens* treatment and had a significant effect on the RFW of the flower head (*p* < 0.001; Figure 1D). There was no significant difference between the mean RFW of cv.s H30 and Fuchsiana at each necking stage and no significant interaction between necking stage and cultivar in terms of RFW (Supplementary Figure 1).

**Figure 2:**
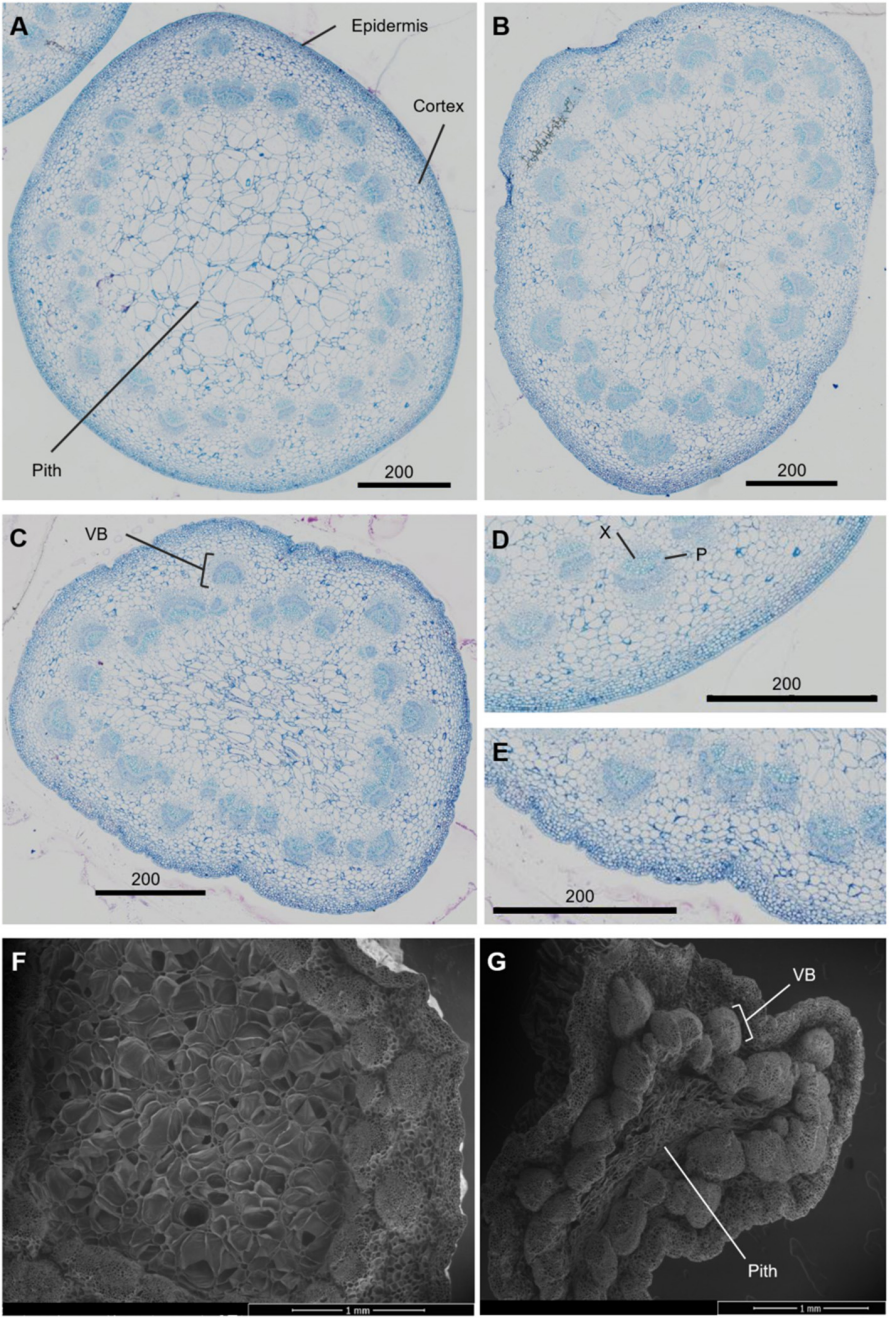
Transverse sections of *Rosa hybrida* cv. H30 peduncles. Light microscopy images stained with toluidine blue at 40x magnification of necking stages: straight **(A)**, <90° **(B)** and ≥90° **(C)**; epidermis of straight **(D)**, and ≥90° peduncles **(E)**. Electron microscopy images of Straight **(F)** and ≥90° **(G)** necking peduncles at 50 x magnification. VB, vascular bundles; X, xylem; P, phloem.

The necking process was divided into two phases: an early phase from straight peduncles to those bending by <90°, and the later stage of bending from <90° to ≥ 90°. Analysis of necking peduncles identified the mid-point of the bend in a necking peduncle to be on average at 19.7 (± 1.2) mm below the flower head, with a mean peduncle length of 97.8 (± 2.2) mm (Supplementary Figure 2). Transverse sections of the predicted mid-point in necking were, therefore, taken at ~20 mm below the flower head for straight peduncles, and <90° peduncles, where the bending point could not easily be identified. Light microscopy sections of necking peduncles showed shrinkage of the pith as necking progresses (Figures 2A–C), with an undulation of the epidermis, clearly seen between ≥90° and straight peduncles (Figures 2D,E). Shrinkage of the pith was also visible in scanning electron microscopy (SEM) images, with greater contrast between the straight and the ≥90° sections (Figures 2F,G).

### Transcriptome Sequencing of Necking Rose Peduncles and Assembly to *Rosa chinensis* Genome Sequence

Necking was recorded under realistic consumer vase life conditions, including commercial flower food, but without induction of the necking. Libraries were prepared using RNA extracted from 20-mm segments of the peduncle centered on the mean mid-point of the necking bend. They were divided into those with a bend of <90° and ≥ 90°, as well as the equivalent region in non-necking stems. Combining data from three biological replicates, a total of 113, 126, and 100 million paired-end reads, respectively, were produced for straight, <90°, and ≥90° sample groups through next-generation sequencing (NGS) (Supplementary Table 2). Alignment of the trimmed reads to the *Rosa chinensis'* Old blush genome with HISAT2 achieved an overall average read alignment rate of 87.6% (Table 1).

**Table 1:** Alignment statistics of transcriptome sequences to the *Rosa chinensis* genome.

**Stage**	**Mean alignment %**	**Mean no. of unique *Rosa chinensis* identifiers**
straight	87.6	33, 589
<90°	87.5	33, 705
>90°	87.5	33, 222

A total of 38,956 unique *Rosa chinensis* genes were identified across all samples, with 34,252 (87.9 %) of the genes appearing in all three of the necking stages, and most genes identified in the straight and <90° stages (Table 1). Of the 38,956 identified genes, 76.2 % had homology to *Arabidopsis thaliana* proteins resulting in 14, 983 unique TAIR10 identifiers, representing 95.3 % of the total TAIR10 proteins with homology to the *Rosa chinensis* genome.

### Identification of Differentially Expressed Genes (DEGs)

A total of 3,647 genes showed significant differential expression across all three comparison groups, based on DESeq2 (*p adjust*. FDR ≤ 0.05). Following differential expression analysis with DESeq2, there were most up- and downregulated genes (3,598 genes) in the comparison between ≥90° and straight necking stages, with a range of 2.2 to −1.79 log_2_ fold change. In contrast, fewest differentially expressed genes (DEGs), only 56, were identified between necking stages <90° and straight. In two of the three comparisons (<90° vs. straight and >90° vs. <90°) more DEGs were down- rather than upregulated (Table 2). Comparing early and later bending, 43 genes were uniquely differentially expressed in the early part of the bending process (from straight to <90°), while many more (794) uniquely changed in expression in the later bending phase (<90° to ≥90°). Only 13 DEGs were shared between early and late bending. The highest number, however, 2,838 genes, only changed significantly in expression between straight peduncles and those bending by ≥90° (Supplementary Figure 3A).

**Table 2:** Differentially expressed genes amongst the three necking stages.

	** <90**°**vs. straight**	**≥90**°**vs. straight**	**≥90°vs. <90°**
Upregulated^#^	17	1,813	353
Downregulated^#^	39	1,785	454
total	56	3,598	807

# *p adjust. <0.05*.

### Pathways and Processes Associated With Necking Peduncles

Of the 3,598 *Rosa chinensis* genes whose expression changed in the >90 *vs*. straight peduncles, 92 % (3,312 genes) were assigned a TAIR10 identifier, equaling a total of 2,865 unique TAIR10 identifiers. Using the TAIR annotations, of the 761 DEGs whose expression changed in more than one of the comparisons (Venn intersections, Supplementary Figure 3A), 234 genes could be assigned a biological process GO term and could be arranged into a biological process heat map (Supplementary Figure 3B). Most changes were consistent in direction of change between the early (<90° compared to straight) and later (≥90° compared to <90°), response with more downregulation than upregulation of expression. Expression of most genes involved with protein phosphorylation, protein ubiquitination, and proteolysis were downregulated, and expression of genes involved with protein de-phosphorylation and protein glycosylation were upregulated in ≥90° necking peduncles. Expression of genes related to metabolic processes (including carbohydrate metabolism and oxidation-reduction), regulation of transcription, and transmembrane transport were both up- and downregulated across the necking stages. However, expression of genes involved with starch biosynthesis, photosynthesis, phosphorelay signal transduction, and metal ion transport was downregulated in ≥90° stage necking peduncles, compared to straight (control) and <90° peduncles. Similarly, expression of genes related to microtubule-based processes, lipid metabolism, and response to stimuli was downregulated in both <90° and ≥90° necking stages in comparison to straight (control) genes. In contrast, the expression of genes related to trehalose biosynthesis was upregulated.

As the ≥90° vs. straight comparison of gene expression yielded the largest number of differentially expressed genes (3,598 genes), this group of genes was further analyzed with the enrichment analysis tools AgriGO v2 (Tian et al., 2017) and Search Tool for Retrieval of Interacting Genes/Proteins (STRING; Szklarczyk et al., 2018) using *Arabidopsis thaliana* (TAIR10) identifiers to detect associated processes and pathways. Of the 2,865 unique TAIR10 annotated genes in this comparison, 762 were upregulated and 1,261 were downregulated (Log_2_ fold change >0.5 and <-0.5, *p adjust*. < 0.05). Parametric analysis of gene set enrichment (PAGE) for biological process with AgriGO v2 identified more significantly down- than upregulated GO terms (Supplementary Figure 4). However, transport (GO:0006810) was found to be a significantly upregulated GO term, with a z score of 4.21 and included a total of 315 transport-associated genes (*p adjust*. < 0.05). Under the parent terms of biological regulation and developmental process: growth (GO:0040007), regulation of cell size (GO:0008361), and cell differentiation (GO:0030154) all showed significant down-regulation, with 86, 61, and 107 associated genes, respectively (*p adjust* < 0.05). Interestingly, the child terms of response to a stimulus: tropism (GO:0009606) and response to abiotic stimulus (GO:0009628) were also found to be significantly downregulated with 23 and 310 identified genes, respectively (Supplementary Figure 4). Of the 23 tropism-related genes identified, 18 were found to be associated with gravitropism and three with phototropism. All three phototropism genes (*NPH3, PHYB*, and *PKS1*) were downregulated, as well as all the tropism genes associated with auxin-activated signaling pathways (*ACB1, ABCB19, AUX1, PIN1*, and *RAC3*) (Supplementary Table 3).

Using singular enrichment analysis (SEA) with STRING (Szklarczyk et al., 2018), and considering the up- and downregulated genes separately, galactose metabolism (GO:ath00052) was identified as a significantly upregulated pathway (FDR < 0.05; Supplementary Table 4A). In contrast, photosynthetic pathways (ath00196 and ath00195) and phenylpropanoid biosynthesis (ath00940) were significantly downregulated (FDR < 0.05; Supplementary Table 4B). However, starch and sucrose metabolism (ath00500), and the more general biosynthesis of secondary metabolites (ath01110) and metabolic pathways (ath01100) included genes that were both significantly up- and downregulated (Supplementary Table 4B).

Parametric analysis of gene set enrichment (PAGE) was also carried out with STRING, using the entire >90° *vs*. straight dataset and corresponding Log_2_ FC values (*p adjust*, < 0.05). This approach revealed water channel activity to be significantly enriched, with an enrichment score of 4.10. Out of the nine aquaporin genes identified, eight showed downregulation (Supplementary Table 5). TIP1-3 was the only water channel gene to be upregulated. However, TIP1-3 also had the lowest mean count of all the aquaporin genes, with an average count of just 34 compared to an average count of over 9,500 for PIP2-7 and almost 1,400 for TIP4-1.

### Differential Expression Analysis of Transcription Factors

**Figure 3:**
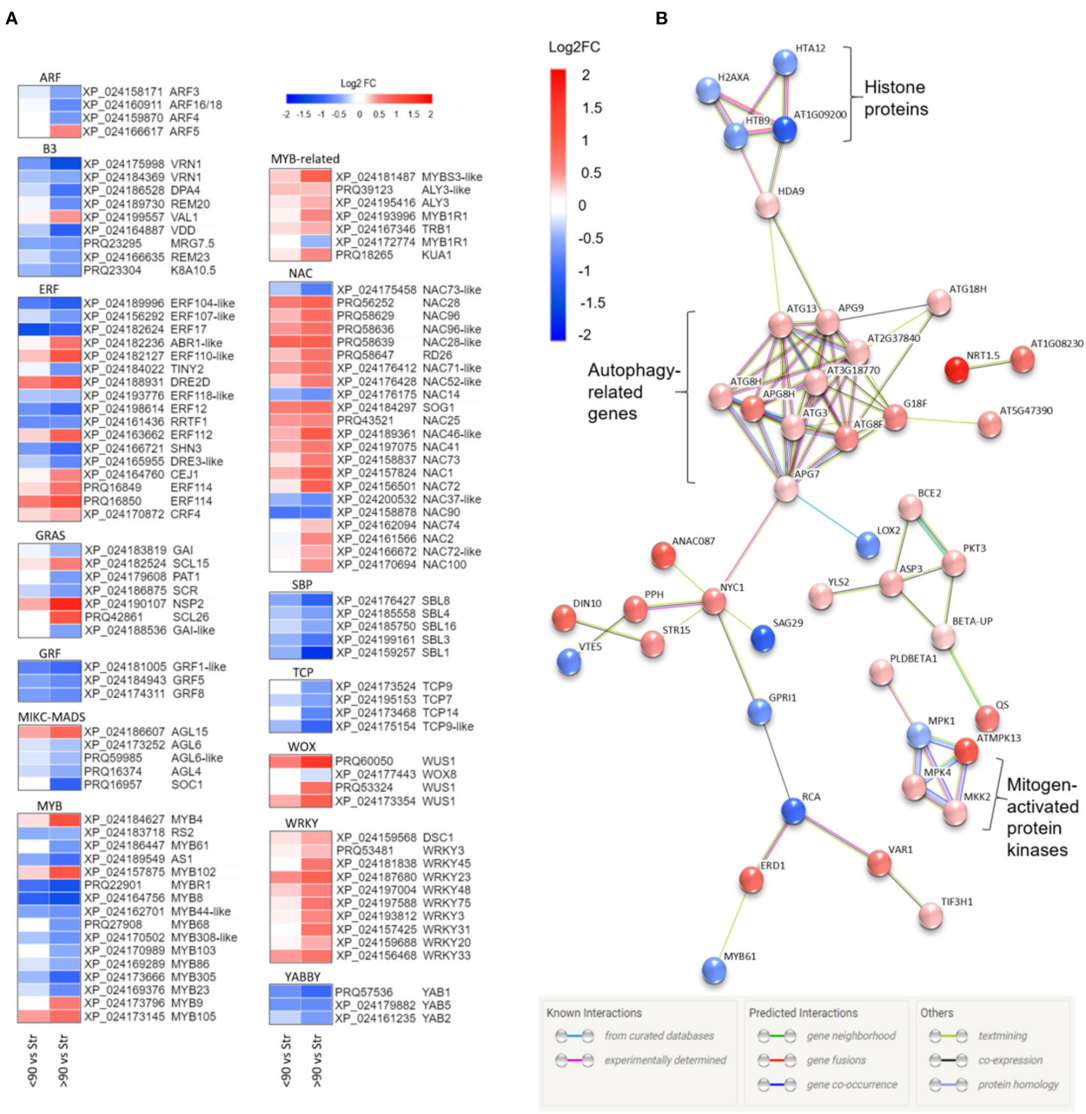
**(A)** Heatmaps of differentially expressed transcription factor families showing Log_2_ fold changes for each *Rosa chinensis* identifier. Up (red) and down (blue) log_2_ fold changes are shown for <90° and ≥90° in relation to straight (control) peduncles. For each data point, there is a significant difference in expression for at least one comparison group (i.e., <90° vs, straight or ≥90° vs, straight). Heatmaps were generated using Morpheus (Morpheus, https://software.broadinstitute.org/morpheus). **(B)** Protein interactions amongst senescence-related genes differentially expressed in ≥90° compared to straight rose peduncles, identified using STRING. Red and blue scales indicate up- and downregulated expression (Log_2_ FC; *p adjust*. < 0.05).

Annotation of the ≥90° vs. straight and <90° vs. straight rose peduncle differentially expressed genes against the transcription factor gene list identified 225 genes from 43 transcription factor families in at least one of the two contrast groups (*p adjust*. <0.05; Supplementary Figure 5). The majority of the families (27) were on average downregulated during necking, while 15 were upregulated. Of particular interest are transcription factor families related to stress and growth responses (Figure 3A). All the genes belonging to the growth-regulating factor (GRF), squamosa promoter binding proteins (SBPs), TCP, YABBY, and Zinc-finger homeodomain (ZF-HD) transcription factor families were downregulated throughout the necking process, and the majority of ARF, B3, and MYB transcription factors were also downregulated. In contrast, all of the differentially expressed genes in the WRKY family, and the majority of genes in the MYB-related, NAC, and WUS homeobox-containing (WOX) transcription factor families showed significant upregulation. Other transcription factor families including ethylene-responsive factor (ERF) and GRAS comprised a mixture of significantly up- and downregulated genes in response to necking.

### Analysis of Senescence-Associated Genes

Based on a comparison to the Uniprot database, 64 of the differentially expressed genes between ≥90° and straight peduncles were identified as senescence-related. Using STRING's multiple protein search, 44 of these genes had connected nodes (Figure 3B). A cluster of eleven autophagy-related genes (ATG) members were significantly upregulated and co-expressed, with many of the connections having been experimentally determined in other systems. A rose gene with homology to histone deacetylase 9 (*HDA9*) was also significantly upregulated, and co-expressed with *APG9* (*ATG9*), linking the ATG cluster to the four histone protein genes *H2AXA, HTB9, HTA12*, and AT1G09200, all of which were significantly downregulated. A cluster of three genes with homology to mitogen-activated protein kinases (MPKs), *MPK1, 4*, and *13*, and an MPK kinase (*MKK2*) were also differentially expressed, with the majority being upregulated. *Histone 3.1* (AT1G09200/ AT5G65360), the bidirectional sugar transporter (*SAG29*), and ribulose carboxylase/oxygenase activase (*RCA*) were the most downregulated genes with connected nodes, (Log_2_ FC −1.16, −1.09, and −0.91 respectively), while transcription factor *TCP9* and mechanosensitive ion channel protein 10 (*MSL10*) (Log_2_ FC −1.02 and −0.99) were also strongly downregulated, but with no known connections to the other senescence genes.

A gene, with homology to a nitrate transporter (*NRT1.5*), was the most upregulated of all the senescence-related genes identified, (Log_2_ FC 1.71); NDR1/HIN1-lie protein 10 (*YLS9*) was the second most upregulated (Log_2_ FC 1.64), however, also had no known connections to the other senescence-associated genes and was, therefore, also not shown.

### Starch and Sugar Metabolism Pathways Were Altered in ≥90° Necking Peduncles

Several changes in expression were noted within the sucrose metabolism pathway (ath00500), when all the up- and downregulated genes with TAIR10 identifiers from the ≥90° *vs*. straight gene set (*p adjust*. <0.05) were included (Supplementary Figure 6, Supplementary Table 6). Genes with homology to *APL1* and *ADG1* encoding the large and small subunits for ADP glucose pyrophosphorylase, (shown as EC: 2.7.7.27 on Supplementary Figure 6) were both downregulated, with an average Log_2_ FC of −0.99. A rose gene with homology to the glucose-starch glycosyltransferase, *GBSS1* (EC: 2.4.1.242) was also downregulated (Log_2_ FC of −0.75). In contrast, genes involved in starch catabolism, α-amylase *AMY2* (EC: 3.2.1.1), and β-amylase *BAM1* (EC: 3.2.1.2) were both upregulated. The β-amylase 2 (*BAM2*), also included in EC: 3.2.1.2, was downregulated, however, there was a mean upregulation of 0.93 (Log_2_ FC) of *BAM1* and *BAM2*. Moreover, the enzymatic activity of BAM2 is much weaker than BAM1 (Fulton et al., 2008).

Glycosidase genes glucan endo-1,3-β-glucosidase genes 1, 2, and 4 (EC: 3.2.1.39) and glycosyl hydrolase 9B1, 9B3, and 9C2 (EC: 3.2.1.4) were downregulated in ≥90° necking peduncles indicating a reduction in 1,3-β-Glucan and cellulose degradation. However, genes involved with the conversion of uridine diphosphate (UDP)-glucose to sucrose and D-fructose (EC: 2.4.1.13), sucrose to D-glucose and D-fructose (EC: 3.2.1.26), D-fructose to D-fructose-6P, and D-glucose to D-glucose-6P (EC: 2.7.1.1) were all upregulated. Together, these lead to a potential increase in both D-fructose-6P and D-glucose-6P for use in glycolysis. Trehalose biosynthetic genes were also upregulated (EC: 2.4.1.15 and EC: 3.1.3.12), including trehalose-phosphatase/synthase 9 and 11, and trehalose-6-phosphate phosphatase G and J. None of the genes encoding trehalase activity (EC: 3.2.1.28) were differentially expressed (*p adjust*. < 0.05) in the ≥90° necking peduncles, indicating a potential increase in trehalose accumulation.

**Figure 4:**
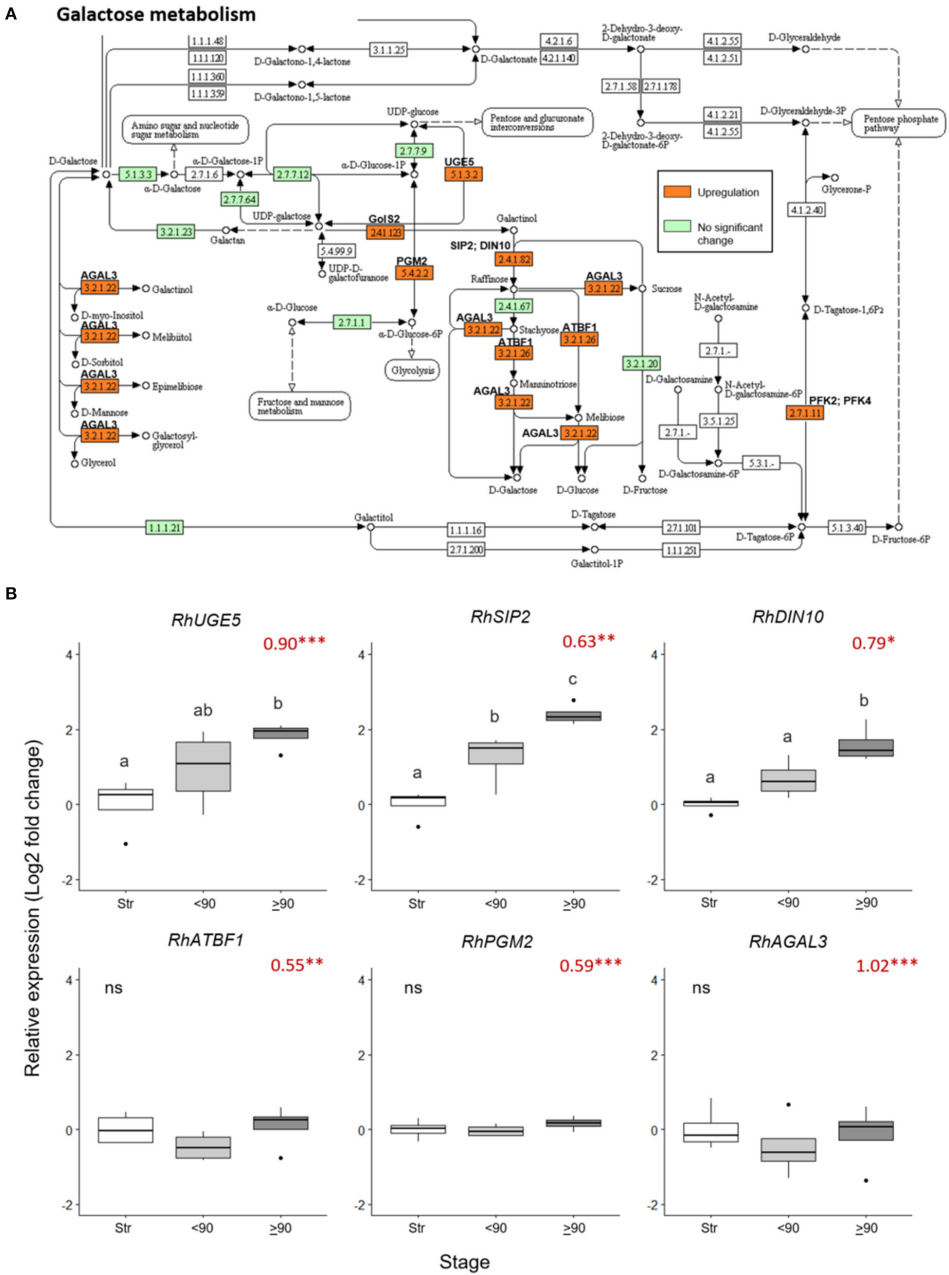
Changes in expression of genes in the galactose metabolism pathway in ≥90° bent compared to straight peduncles. **(A)** Pathway adapted from KEGG (rcn00052; Kanehisa, 2017) and showing EC codes for enzymes. Significantly upregulated genes (Log_2_ FC > 0.5 and a *p adjust*. value < 0.05) are shown in orange. EC codes shown in green represent enzymes within the *R. chinensis* genome with no significant change and EC codes in white represent enzymes not identified in *R. chinensis*. **(B)** qPCR relative expression of six galactose-related genes. Pfaffle analysis is relative to RhGACPC2 expression using straight peduncles as the control. Different letters indicate significant differences (*p* < 0.05) based on one way ANOVA with a Tukey *post hoc* test and ‘ns' indicates no significant difference. Red numbers indicate RNAseq Log2 FC (in brackets on the y-axis), where asterisks are *** *p adjust*. < 0.001, ** < 0.01, * < 0.05.

Nine galactose metabolism-related genes were significantly upregulated (> 0.5 Log_2_ FC, *p adjust* < 0.05) in the ≥90° necking peduncles, compared to straight peduncles (Figure 4A, Supplementary Table 7). The expression pattern of three of these genes, *RhUGE5* (EC: 5.1.3.2), *RhSIP2* (EC: 2.4.1.82), and *RhDIN10* (EC: 2.4.1.82) was confirmed by real-time PCR (Figure 4B), inducing necking using *Pseudomonas fluorescens*. This verified that consumer vase life conditions and the induced necking were consistent in expression changes elicited. All three genes showed a progressive increase in expression with peduncle necking. The mean expression of *RhATBF1, RhPGM2*, and *RhAGAL3* was also higher in ≥90° necking than in straight or <90° necked peduncles, however, the differences were not significant (*p* < 0.05).

### Phenylpropanoid Biosynthesis Was Downregulated With Necking

**Figure 5:**
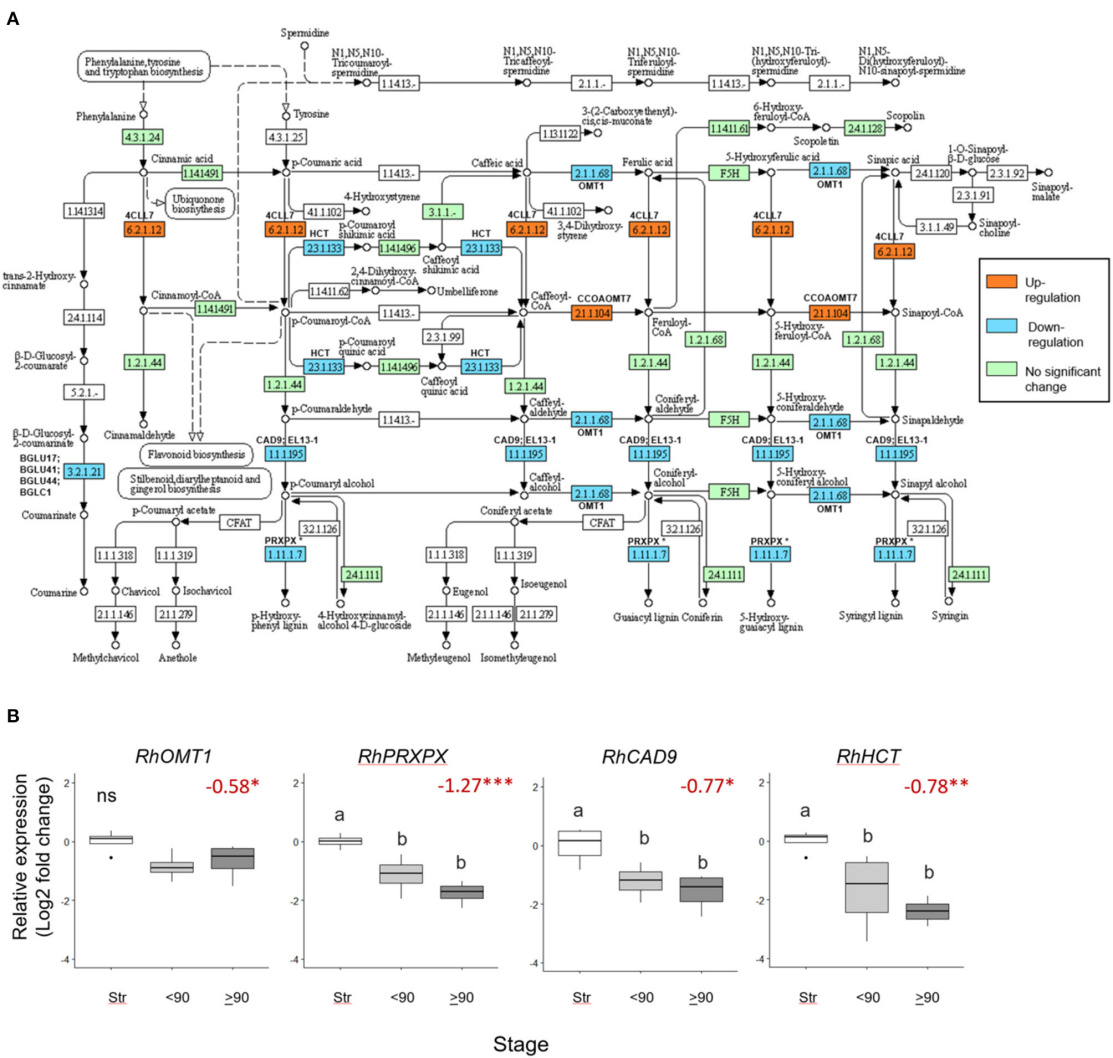
Differentially expressed phenylpropanoid biosynthesis genes in ≥90 ° bent compared to straight peduncles. **(A)** pathway analysis: green EC numbers represent genes present in the *Rosa chinensis* genome, blue, downregulated, and orange upregulated genes (< −0.5 Log_2_ FC, *p adjust*. < 0.05). Note that EC1.11.1.7 has nine associated downregulated peroxidase genes, of which PRXPX has the highest mean count (other genes not shown). Pathway adapted and annotated from KEGG (pathway: rcn00940). **(B)** qPCR relative expression of four phenylpropanoid biosynthesis genes. Pfaffle analysis is relative to *RhGACPC2* expression using straight peduncles as the control. Different letters indicate significant differences (*p* < 0.05) based on one way ANOVA with a Tukey *post hoc* test and ‘ns' indicates no significant difference. Red numbers indicate RNAseq Log_2_ FC (in brackets on the y-axis) where asterisks are*** *p adjust*. < 0.001, ** < 0.01, * < 0.05.

Seventeen phenylpropanoid biosynthesis genes were significantly downregulated (>0.5 Log_2_ FC, *p adjust* <0.05) in ≥90° necked, compared to straight peduncles (Figure 5A), associated with five EC codes. To verify the RNAseq data expression of four genes: *RhOMT1, RhPXPX42, RhCAD9*, and *RhHCT* was also analyzed by real-time PCR (Supplementary Table 7). Where several rose genes were associated with a single EC point, the representative was selected as the gene with the highest expression (based on the mean count).

Expression of *RhPXPX42, RhCAD9*, and *RhHCT* genes was significantly downregulated between ≥90° necked and straight peduncles in both the RNA seq. and qPCR datasets, and, indeed, real-time PCR also revealed a significant downregulation between straight and <90° necked peduncles. Mean *RhOMT1* expression was also lower in <90° compared to straight peduncles, however, the change was not significant (*p* < 0.05) in the rt-PCR analysis. Thus, three out of the four genes analyzed by rt-PCR mirrored the downregulation seen with RNAseq as necking progressed (Figure 5B).

## Discussion

Vase life varies across rose cultivars and is dependent on an interaction amongst genotype, pre-harvest, and post-harvest conditions (Fanourakis et al., 2013). The short vase-lives of “Akito” and “H3O” noted here seem to relate to their water loss following dry transportation and ability to rehydrate. In the case of “Akito”, this has been linked to the epidermal structure, which favors water loss during drought (Matsushima et al., 2010). Necking or “bent neck”, although not the only deterioration that results in termination of vase life (Fanourakis et al., 2013), is a significant problem. “Akito” was described as having low necking resistance (Matsushima et al., 2010) and, indeed, here, the majority of vase life termination in both “Akito” and “H3O” was due to necking. The addition of *Pseudomonas fluorescens* to the vase water resulted in consistent necking and irrespective of the cultivar, necking was associated with a progressive reduction in flower head fresh weight. This suggests that the necking reduces water transport to the flower resulting in an increasing angle of bending. This was confirmed through microscopy showing shrunken collapsed cells, in line with previous work using cold neutron radiography on the peduncle during necking (Matsushima et al., 2010), which, in this case, was induced by dehydration stress. This further indicates that similar cellular effects are induced during necking with or without microbial addition.

The large number of DEGs identified amongst different necking stages, including upregulated genes, confirms that necking is an active process. Fold differences in expression suggest subtle changes or changes occurring only in a small number of the peduncle cells. The far greater number of DEGs in the >90° vs. <90° compared to the <90° vs. straight comparison and the small number of DEGs shared suggests that the two phases of necking are distinct from the later part of the bending, thus, being more important in terms of metabolic changes. However, as found with gerbera (Ge et al., 2019), the majority of the genes that changed in expression in the early bending phase also changed in the later phase indicating that processes activated early were sustained throughout necking. The downregulation of genes related to growth and cell differentiation, and response to stimuli is consistent with damage to the tissue imposed by the necking. Contributing to this is the downregulation of transcription factors that are primarily associated with plant growth and development including *GRF, MIKC-MADS, MYB, SBP, TCP*, and *YABBY* (Gonzalez, 2015).

Both photosynthesis and starch biosynthesis were downregulated, and these two pathways are closely linked, with starch degradation often increasing in response to a reduction in photosynthesis to meet cellular energy requirements (Zanella et al., 2016). *ANAC087* (*R. chinensis NAC46-like*) positively regulates senescence in Arabidopsis leaves by promoting the expression of genes associated with chlorophyll catabolism, including non-yellow coloring 1 (*NYC1*) and pheophytinase (*PPH*) (Oda-Yamamizo et al., 2016). All three genes showed significant increases in expression in >90° necking peduncles, correlating with the significant downregulation of photosynthesis. A more detailed examination of the pathway found downregulation of genes related to several starch biosynthetic enzymes in ≥90° necking compared to straight peduncles, and upregulation of genes related to starch breakdown: α-amylase (*AMY2*) and β-amylase (*BAM1*). Under normal conditions, BAM1 is not the primary β-amylase responsible for starch degradation, however, expression has been shown to increase following osmotic stress and is essential for proline accumulation in *A. thaliana* to provide osmoprotection and protect against oxidative damage (Zanella et al., 2016) An increase in amylase gene expression was also noted in *Populus* stems (Secchi et al., 2011) and grapevine leaf petioles (Perrone et al., 2012) in response to embolism. It was hypothesized in *Populus* that the sugars released might also have an osmotic role in drawing water back into the embolised cells. However, unlike in *Populus* and grapevine, most rose aquaporin genes were downregulated during necking, hence, it is more likely that the sugars released from the starch breakdown are being used as an energy source. The increase in expression of trehalose metabolism-related genes is, nevertheless, consistent with the necking peduncle, experiencing and protecting against osmotic stress since trehalose is accumulated in response to dehydration to stabilize proteins and membranes (Figueroa and Lunn, 2016). Genes related to galactose metabolism were also upregulated with peduncle necking, and one of the products of this pathway is raffinose. Raffinose production has been shown to increase in response to dehydration along with a downregulation in photosynthesis (Farrant et al., 2015) and is important in osmoprotection and reactive oxygen species (ROS) scavenging (ElSayed et al., 2014). The upregulation of three genes relating to raffinose production was confirmed by rt-PCR: *UGE5* catalyses the synthesis of UDP-galactose from UDP-glucose, and both catalyse the synthesis of raffinose from galactinol and sucrose (Nishizawa et al., 2008). This is consistent with rose peduncles experiencing and responding to a water deficit.

Another pathway that was downregulated in the necking peduncles was phenylpropanoid metabolism, which was also over-represented in gerbera bending (Ge et al., 2019). This is of relevance in its potential involvement in responses to xylem blockage by microorganisms, since necking could be reliably induced with *Pseudomonas fluorescens*. In other plants, upregulation of phenylpropanoid biosynthesis genes involved with lignin biosynthesis, including shikimate o-hydroxy-cinnamoyltransferase (HCT), was seen in response to bacterial and fungal plant pathogens (Patel et al., 2017). The reduction in *RhCAD9, RhPRXPX*, and *RhHCT* expressions seen here, confirmed by rt-PCR, strongly indicates that activation of lignin biosynthesis in response to microorganism challenges is not part of the mechanism of necking. *MYB103*, with *ANAC073*, induces expression of secondary cell wall biosynthesis genes, including cellulose, hemicellulose, and lignin biosynthetic genes in Arabidopsis (Zhong et al., 2008). Both a *RhMYB103* and an *RhNAC73*-like gene were also downregulated here, consistent with the downregulation of this regulatory pathway. Lignin biosynthesis was also downregulated during gerbera stem bending (Ge et al., 2019). Although in gerbera, bending is not thought to involve microorganisms, so it could be a general response to dehydration.

The change in expression of transcription factors, such as the upregulation of most *NAC* and all *WRKY* genes, is consistent with dehydration-induced senescence in necking peduncles and was also found in other stems undergoing embolisms (Secchi et al., 2011; Ge et al., 2019). However, the *NF-Y* transcription factors, implicated in drought stress responses, were downregulated in rose necking, in contrast to gerbera stem bending, where they were upregulated (Ge et al., 2019). Orthologs of *ANAC72, ANAC002*, and *ANAC100* were also significantly upregulated in *Pyrus Bretschneider* (white pear), following drought treatment (Gong et al., 2019), and *ANAC072* (*RD26)* regulates increased expression of drought-responsive genes and negatively regulates plant growth in Arabidopsis (Ye et al., 2017). Moreover, *ANAC072* and *ANAC002* (*ATAF1*) are positive regulators of senescence. *ANAC002* was found to regulate ATG and *ANAC072* was found to induce protein degradation (including chloroplast degradation) and increase expression of *AMY* and *SUS* starch and sucrose catabolism genes (Kamranfar et al., 2018). However, *WRKY70* a leaf senescence-associated transcription factor also found in the gerbera stem bending transcriptome (Ge et al., 2019) was not upregulated in the necking peduncles, suggesting that a different senescence program is activated in rose bending/necking peduncles compared to leaves and gerbera stem bending. Several ATGs were upregulated during rose necking, including ATG8 isoform genes (*ATG8F* and *ATG8H*), which are central proteins involved in autophagy and abiotic stress responses, including osmotic stress, as well as *ATG13a, ATG13b*, and *ATG11* autophagy genes which, are involved in responses to carbon and nitrogen starvation (Wang et al., 2017). Moreover, trehalose has been shown to initiate controlled autophagy to protect cells in abiotic stress conditions (Williams et al., 2015), which may provide a link to changes in sugar metabolism seen in the necking peduncles. The upregulation of the *ABR1-like* ERF transcription factor also supports a drought response since in Arabidopsis *ABR1*, is thought to be involved in drought stress responses (Pandey et al., 2005). However, in common with gerbera stem bending (Ge et al., 2019), there was no strong evidence for upregulation of ethylene-responsive genes, indicating that ethylene is unlikely to be a major regulator of rose necking.

The finding that most tropism-related genes were downregulated is of relevance in considering necking as a type of stem bending. Tropic responses to gravity and light are active processes involving growth (Harmer and Brooks, 2018) and, hence, are unlikely to be involved in rose necking. However, the disruption of gravitropic responses in the peduncle, especially the downregulation of *SHOOT GRAITROPISM9* (*SGR9*), that modulates amyloplast dynamics, and, hence, gravity perception might be relevant in determining the precise point in the peduncle where necking occurs, as this is related to amyloplast displacement (Weise et al., 2000).

The upregulation of several known senescence-associated genes also supports a mechanism of drought-induced senescence. Nitrate transporter *NRT1.5* was the most upregulated gene associated with the term senescence in >90° peduncles compared to straight controls and is upregulated in senescent Arabidopsis leaves to remobilise nitrates and ammonium (Hav et al., 2016). *GAT1*, a gamma-aminobutyric acid (GABA) transporter, was also significantly upregulated in >90° necking peduncles and is expressed in response to elevated levels of GABA in Arabidopsis stress-induced senescence (Meyer et al., 2006). In contrast, *SAG29* was one of the most downregulated senescence identified genes in >90° necking peduncles. *SAG29* expression is induced in Arabidopsis by osmotic stress in an abscisic acid-dependent manner (Seo et al., 2010). However, SAG29 is thought to be involved with later stages of senescence and may regulate cell death. Therefore, reduced expression of *SAG29* in necking peduncles could indicate that cells at this stage are at an early stage of senescence. Alternatively, the reduced expression of *SAG29* may indicate a lack of ABA involvement in >90° necking stage processes as supported by the small number of abscissic acid (ABA)-related genes amongst the DEGs. This would indicate a different mechanism, to that found in gerbera, where ABA was identified as an important regulator for stem bending (Ge et al., 2019). However, the involvement of ABA in recovery from embolisms may be related to transpiration status as an increase in ABA was only found in grapevine leaf petioles (Perrone et al., 2012) in conditions of high transpiration.

In conclusion, based on the transcriptomic and microscopy data presented, a model can be developed for rose necking, in which xylem blockage results in drought and osmotic protection responses. However, as necking progresses, autophagic-like cellular senescence is induced, thus, ending in cell collapse. Based on this model, treatments and breeding strategies need to be aimed at improving the resilience of the xylem to blockage and avoid the initiation of the necking cellular collapse.

## Data Availability Statement

The datasets presented in this study can be found in online repositories. The names of the repository/repositories and accession number(s) can be found below: https://www.ncbi.nlm.nih.gov/sra/PRJNA783053.

## Author Contributions

BL conducted the experimental work and drafted the manuscript. MC assisted with data analysis. HR and AS designed the project and co-wrote the manuscript. All authors revised the manuscript. All authors contributed to the article and approved the submitted version.

## Funding

MC and BL were funded by an iCASE BBSRC studentships in collaboration respectively with Chrysal and Flamingo.

## Conflict of Interest

The authors declare that the research was conducted in the absence of any commercial or financial relationships that could be construed as a potential conflict of interest.

## Publisher's Note

All claims expressed in this article are solely those of the authors and do not necessarily represent those of their affiliated organizations, or those of the publisher, the editors and the reviewers. Any product that may be evaluated in this article, or claim that may be made by its manufacturer, is not guaranteed or endorsed by the publisher.

## Supplementary Material

Supplementary Table 1 All primer sequences used for PCR.

Supplementary Table 2 Overview of RNAseq data sets: quality and number of reads.

Supplementary Table 3 Tropism-related genes whose expression changed significantly between ≥90° necking and straight peduncles.

Supplementary Table 4 Significantly upregulated **(A)** and downregulated **(B)** pathways in the ≥90° vs. straight comparison of gene expression using singular enrichment analysis (SEA).

Supplementary Table 5 Aquaporin genes whose expression changed significantly between ≥90° necking and straight peduncles.

Supplementary Table 6 **(A)** Significant changes in expression in individual starch and sucrose metabolism pathway genes and **(B)** count weighted mean FC per EC number, in the ≥90° vs. straight comparison of gene expression using Kyoto Encyclopedia of Genes and Genomes (KEGG).

Supplementary Table 7 RNA sequencing output from differential expression analysis for galactose metabolism and phenylpropanoid pathway genes chosen for qPCR analysis.

Supplementary Figure 1 Relative fresh weights of ‘H30' and ‘Fuchsiana' flower stems at three necking stages.

Supplementary Figure 2 Mean peduncle length and points of necking in ‘H30'.

Supplementary Figure 3 Differential gene expression amongst necking stages in cv. H3O peduncles.

Supplementary Figure 4 Gene ontology (GO) terms identified using AgriGO v2.

Supplementary Figure 5 Heatmap of differentially expressed transcription factor families.

Supplementary Figure 6 Changes in expression of genes in the starch and sucrose metabolism pathway.
