# Supplementary material for: Peduncle Necking in Rosa hybrida Induces Stress-Related Transcription Factors, Upregulates Galactose Metabolism, and Downregulates Phenylpropanoid Biosynthesis Genes
Source: Front Plant Sci. 2022 Apr 18;13:874590. doi: 10.3389/fpls.2022.874590 (PMC9062881; doi:10.3389/fpls.2022.874590)
Supplement: Supplementary Table 1 — All primer sequences used for PCR. [file Presentation_1.PPTX]

## Slide 1
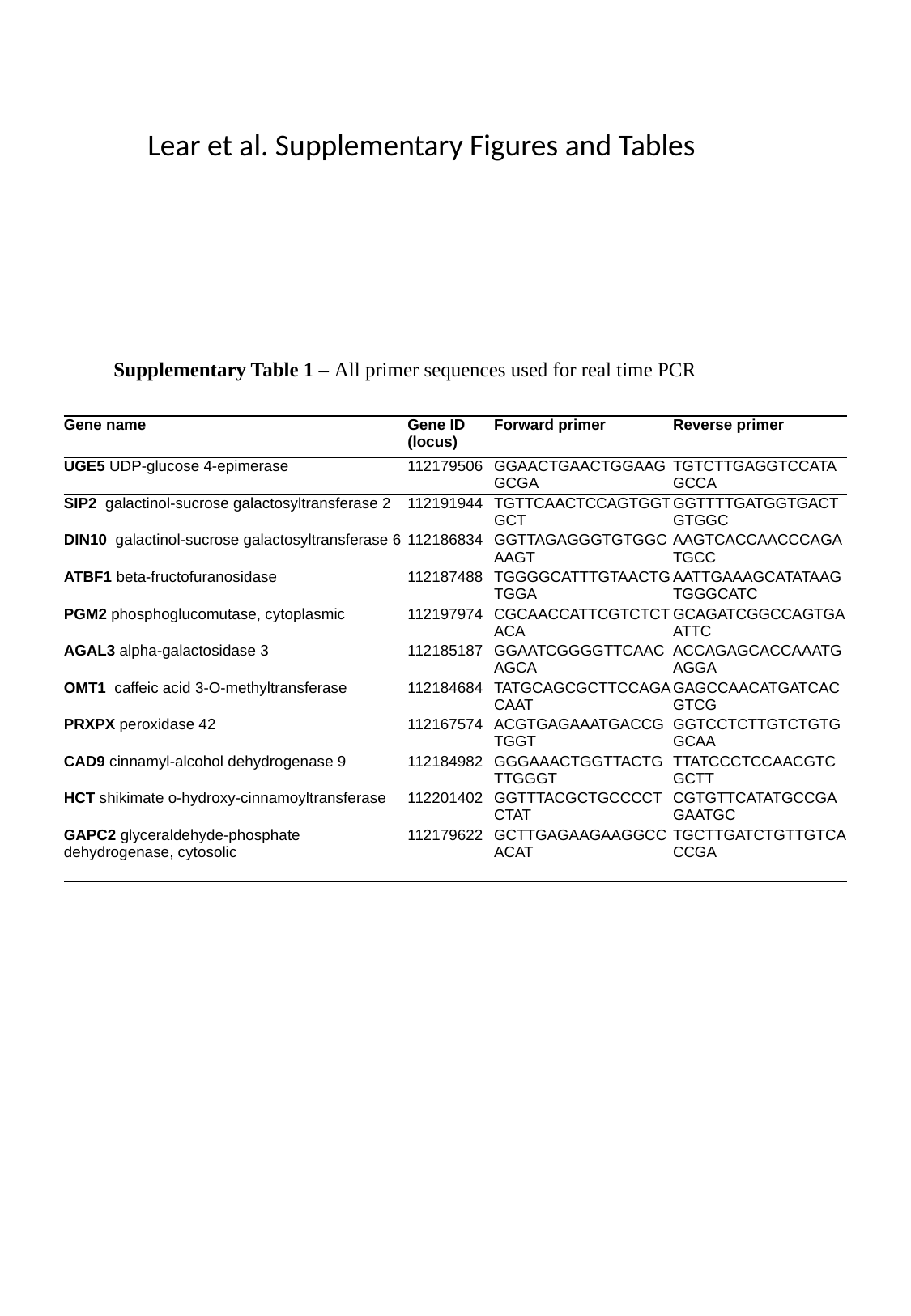

Lear et al. Supplementary Figures and Tables
Supplementary Table 1 – All primer sequences used for real time PCR
| Gene name | Gene ID (locus) | Forward primer | Reverse primer |
| --- | --- | --- | --- |
| UGE5 UDP-glucose 4-epimerase | 112179506 | GGAACTGAACTGGAAGGCGA | TGTCTTGAGGTCCATAGCCA |
| SIP2 galactinol-sucrose galactosyltransferase 2 | 112191944 | TGTTCAACTCCAGTGGTGCT | GGTTTTGATGGTGACTGTGGC |
| DIN10 galactinol-sucrose galactosyltransferase 6 | 112186834 | GGTTAGAGGGTGTGGCAAGT | AAGTCACCAACCCAGATGCC |
| ATBF1 beta-fructofuranosidase | 112187488 | TGGGGCATTTGTAACTGTGGA | AATTGAAAGCATATAAGTGGGCATC |
| PGM2 phosphoglucomutase, cytoplasmic | 112197974 | CGCAACCATTCGTCTCTACA | GCAGATCGGCCAGTGAATTC |
| AGAL3 alpha-galactosidase 3 | 112185187 | GGAATCGGGGTTCAACAGCA | ACCAGAGCACCAAATGAGGA |
| OMT1 caffeic acid 3-O-methyltransferase | 112184684 | TATGCAGCGCTTCCAGACAAT | GAGCCAACATGATCACGTCG |
| PRXPX peroxidase 42 | 112167574 | ACGTGAGAAATGACCGTGGT | GGTCCTCTTGTCTGTGGCAA |
| CAD9 cinnamyl-alcohol dehydrogenase 9 | 112184982 | GGGAAACTGGTTACTGTTGGGT | TTATCCCTCCAACGTCGCTT |
| HCT shikimate o-hydroxy-cinnamoyltransferase | 112201402 | GGTTTACGCTGCCCCTCTAT | CGTGTTCATATGCCGAGAATGC |
| GAPC2 glyceraldehyde-phosphate dehydrogenase, cytosolic | 112179622 | GCTTGAGAAGAAGGCCACAT | TGCTTGATCTGTTGTCACCGA |

## Slide 2
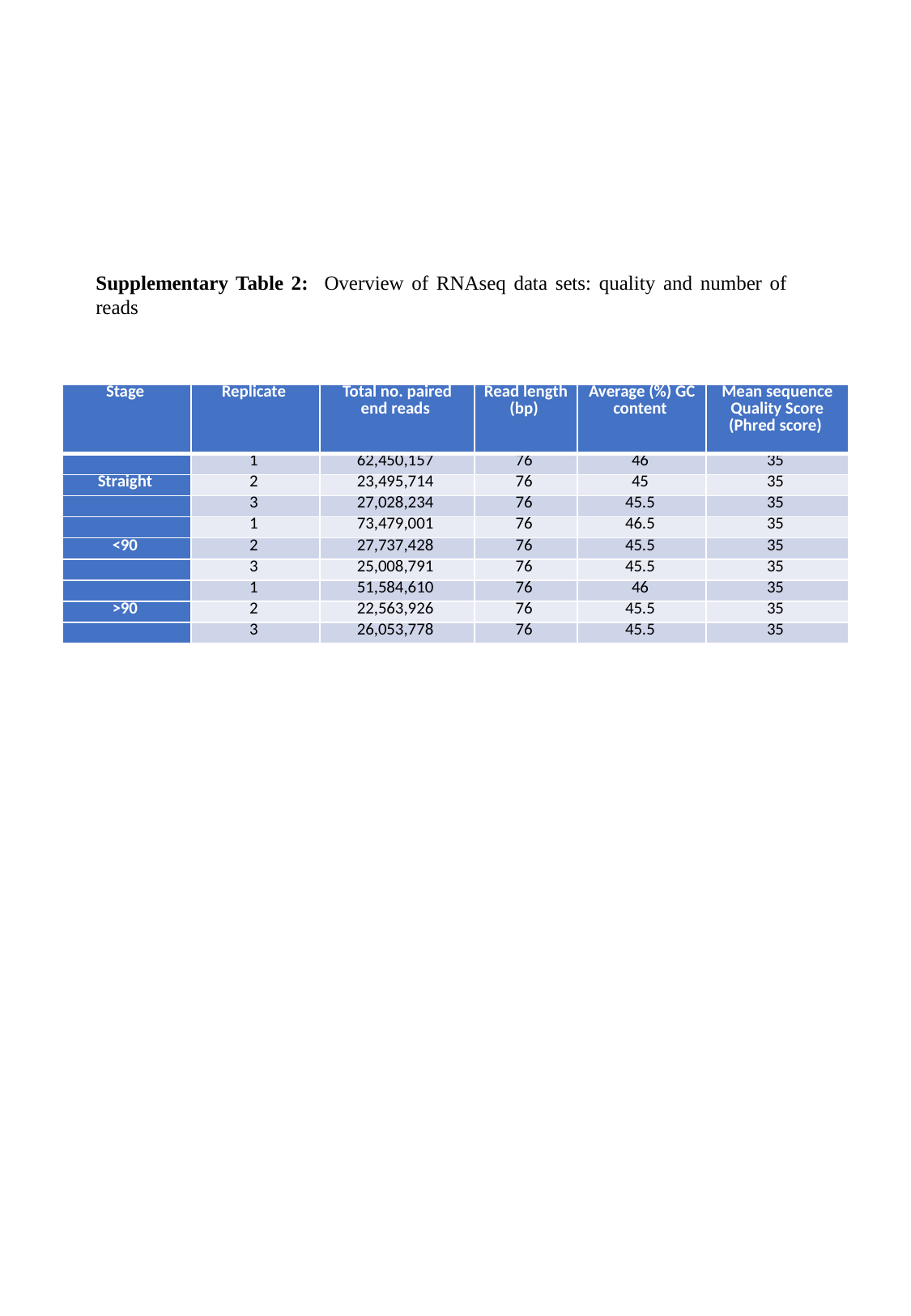

Supplementary Table 2: Overview of RNAseq data sets: quality and number of reads
| Stage | Replicate | Total no. paired end reads | Read length (bp) | Average (%) GC content | Mean sequence Quality Score (Phred score) |
| --- | --- | --- | --- | --- | --- |
| | 1 | 62,450,157 | 76 | 46 | 35 |
| Straight | 2 | 23,495,714 | 76 | 45 | 35 |
| | 3 | 27,028,234 | 76 | 45.5 | 35 |
| | 1 | 73,479,001 | 76 | 46.5 | 35 |
| <90 | 2 | 27,737,428 | 76 | 45.5 | 35 |
| | 3 | 25,008,791 | 76 | 45.5 | 35 |
| | 1 | 51,584,610 | 76 | 46 | 35 |
| >90 | 2 | 22,563,926 | 76 | 45.5 | 35 |
| | 3 | 26,053,778 | 76 | 45.5 | 35 |

## Slide 3
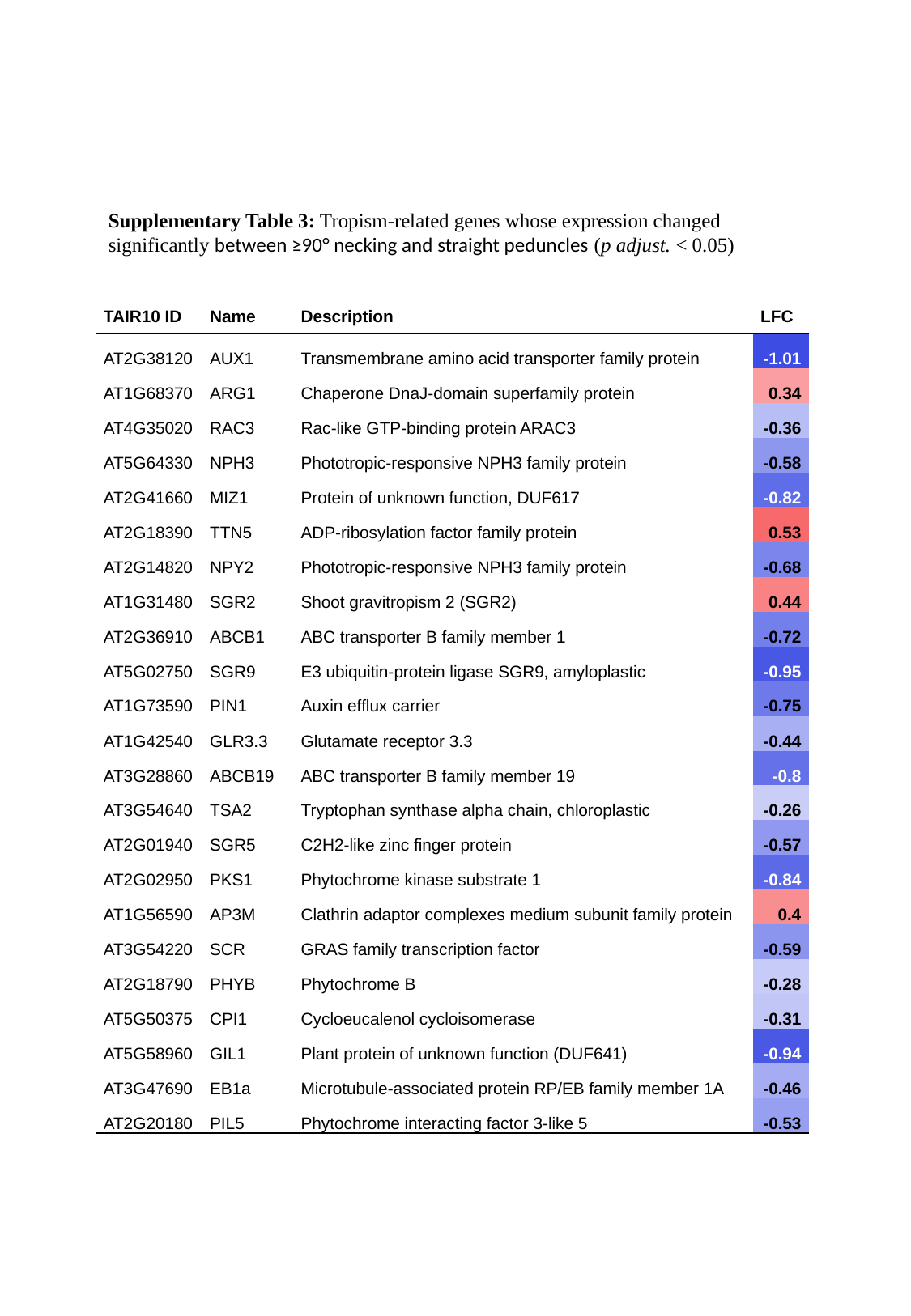

Supplementary Table 3: Tropism-related genes whose expression changed significantly between ≥90° necking and straight peduncles (p adjust. < 0.05)
| TAIR10 ID | Name | Description | LFC |
| --- | --- | --- | --- |
| AT2G38120 | AUX1 | Transmembrane amino acid transporter family protein | -1.01 |
| AT1G68370 | ARG1 | Chaperone DnaJ-domain superfamily protein | 0.34 |
| AT4G35020 | RAC3 | Rac-like GTP-binding protein ARAC3 | -0.36 |
| AT5G64330 | NPH3 | Phototropic-responsive NPH3 family protein | -0.58 |
| AT2G41660 | MIZ1 | Protein of unknown function, DUF617 | -0.82 |
| AT2G18390 | TTN5 | ADP-ribosylation factor family protein | 0.53 |
| AT2G14820 | NPY2 | Phototropic-responsive NPH3 family protein | -0.68 |
| AT1G31480 | SGR2 | Shoot gravitropism 2 (SGR2) | 0.44 |
| AT2G36910 | ABCB1 | ABC transporter B family member 1 | -0.72 |
| AT5G02750 | SGR9 | E3 ubiquitin-protein ligase SGR9, amyloplastic | -0.95 |
| AT1G73590 | PIN1 | Auxin efflux carrier | -0.75 |
| AT1G42540 | GLR3.3 | Glutamate receptor 3.3 | -0.44 |
| AT3G28860 | ABCB19 | ABC transporter B family member 19 | -0.8 |
| AT3G54640 | TSA2 | Tryptophan synthase alpha chain, chloroplastic | -0.26 |
| AT2G01940 | SGR5 | C2H2-like zinc finger protein | -0.57 |
| AT2G02950 | PKS1 | Phytochrome kinase substrate 1 | -0.84 |
| AT1G56590 | AP3M | Clathrin adaptor complexes medium subunit family protein | 0.4 |
| AT3G54220 | SCR | GRAS family transcription factor | -0.59 |
| AT2G18790 | PHYB | Phytochrome B | -0.28 |
| AT5G50375 | CPI1 | Cycloeucalenol cycloisomerase | -0.31 |
| AT5G58960 | GIL1 | Plant protein of unknown function (DUF641) | -0.94 |
| AT3G47690 | EB1a | Microtubule-associated protein RP/EB family member 1A | -0.46 |
| AT2G20180 | PIL5 | Phytochrome interacting factor 3-like 5 | -0.53 |

## Slide 4
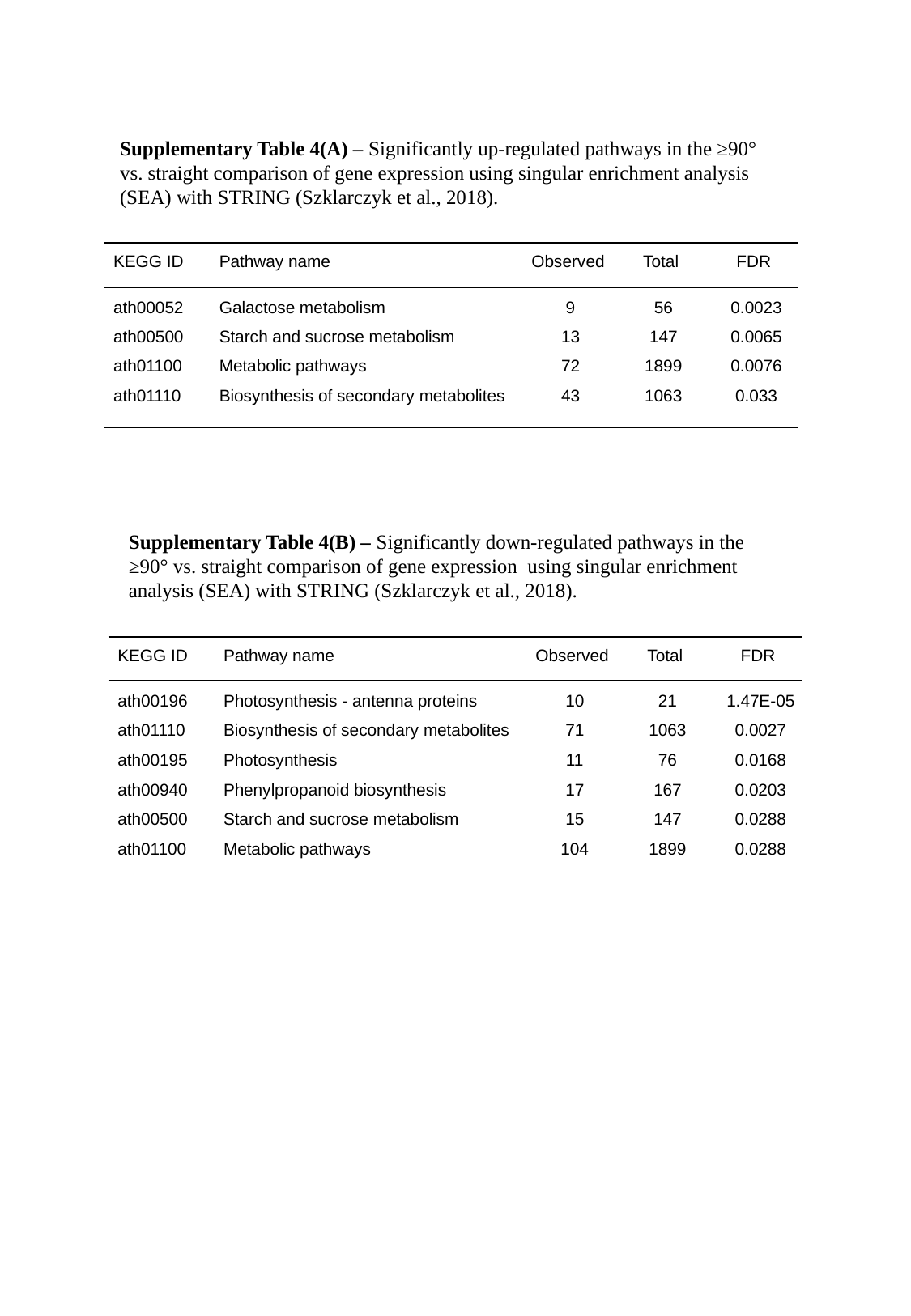

Supplementary Table 4(A) – Significantly up-regulated pathways in the ≥90° vs. straight comparison of gene expression using singular enrichment analysis (SEA) with STRING (Szklarczyk et al., 2018).
| KEGG ID | Pathway name | Observed | Total | FDR |
| --- | --- | --- | --- | --- |
| ath00052 | Galactose metabolism | 9 | 56 | 0.0023 |
| ath00500 | Starch and sucrose metabolism | 13 | 147 | 0.0065 |
| ath01100 | Metabolic pathways | 72 | 1899 | 0.0076 |
| ath01110 | Biosynthesis of secondary metabolites | 43 | 1063 | 0.033 |
| | | | | |
Supplementary Table 4(B) – Significantly down-regulated pathways in the ≥90° vs. straight comparison of gene expression using singular enrichment analysis (SEA) with STRING (Szklarczyk et al., 2018).
| KEGG ID | Pathway name | Observed | Total | FDR |
| --- | --- | --- | --- | --- |
| ath00196 | Photosynthesis - antenna proteins | 10 | 21 | 1.47E-05 |
| ath01110 | Biosynthesis of secondary metabolites | 71 | 1063 | 0.0027 |
| ath00195 | Photosynthesis | 11 | 76 | 0.0168 |
| ath00940 | Phenylpropanoid biosynthesis | 17 | 167 | 0.0203 |
| ath00500 | Starch and sucrose metabolism | 15 | 147 | 0.0288 |
| ath01100 | Metabolic pathways | 104 | 1899 | 0.0288 |
| | | | | |

## Slide 5
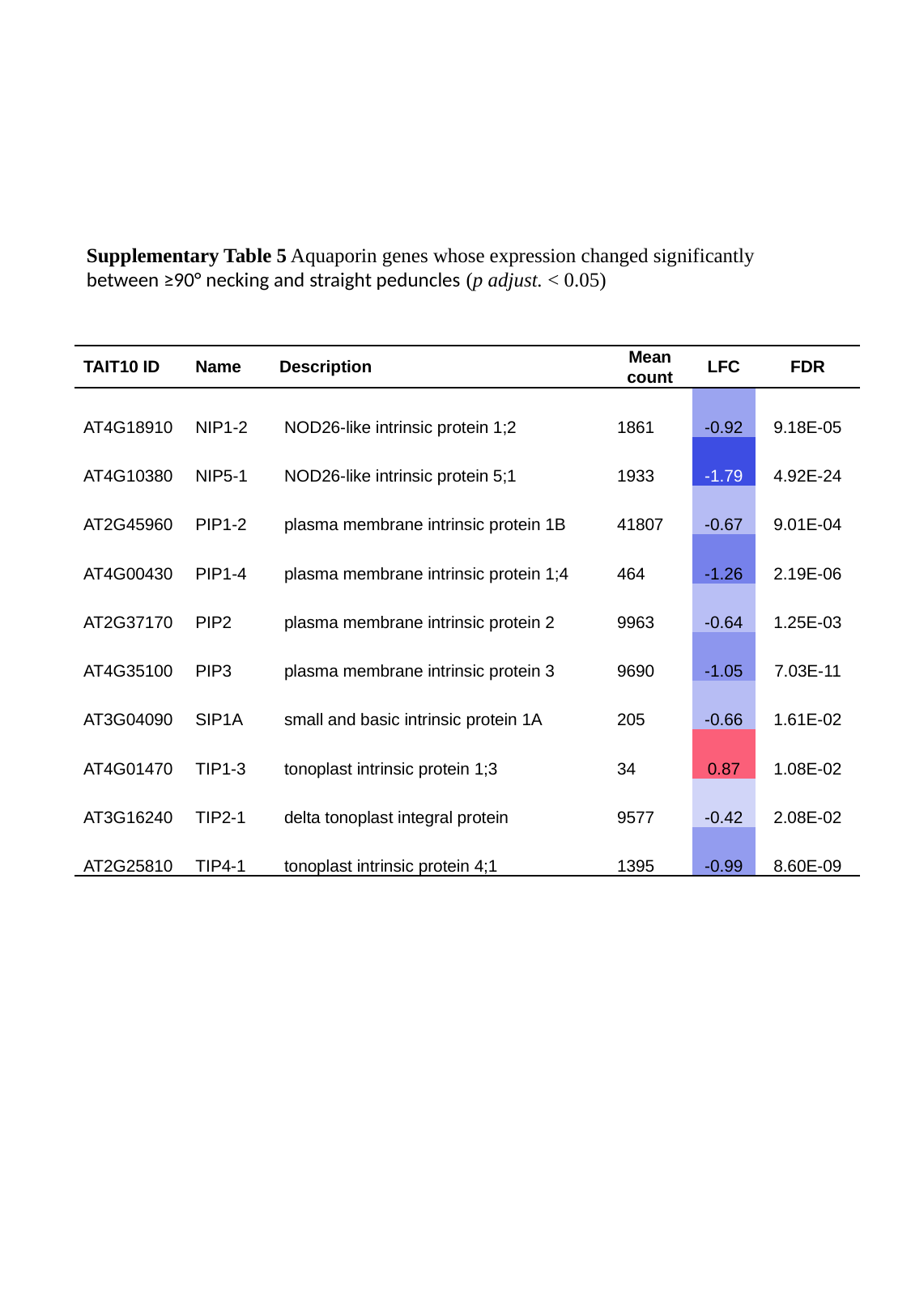

Supplementary Table 5 Aquaporin genes whose expression changed significantly between ≥90° necking and straight peduncles (p adjust. < 0.05)
| TAIT10 ID | Name | Description | Mean count | LFC | FDR |
| --- | --- | --- | --- | --- | --- |
| AT4G18910 | NIP1-2 | NOD26-like intrinsic protein 1;2 | 1861 | -0.92 | 9.18E-05 |
| AT4G10380 | NIP5-1 | NOD26-like intrinsic protein 5;1 | 1933 | -1.79 | 4.92E-24 |
| AT2G45960 | PIP1-2 | plasma membrane intrinsic protein 1B | 41807 | -0.67 | 9.01E-04 |
| AT4G00430 | PIP1-4 | plasma membrane intrinsic protein 1;4 | 464 | -1.26 | 2.19E-06 |
| AT2G37170 | PIP2 | plasma membrane intrinsic protein 2 | 9963 | -0.64 | 1.25E-03 |
| AT4G35100 | PIP3 | plasma membrane intrinsic protein 3 | 9690 | -1.05 | 7.03E-11 |
| AT3G04090 | SIP1A | small and basic intrinsic protein 1A | 205 | -0.66 | 1.61E-02 |
| AT4G01470 | TIP1-3 | tonoplast intrinsic protein 1;3 | 34 | 0.87 | 1.08E-02 |
| AT3G16240 | TIP2-1 | delta tonoplast integral protein | 9577 | -0.42 | 2.08E-02 |
| AT2G25810 | TIP4-1 | tonoplast intrinsic protein 4;1 | 1395 | -0.99 | 8.60E-09 |

## Slide 6
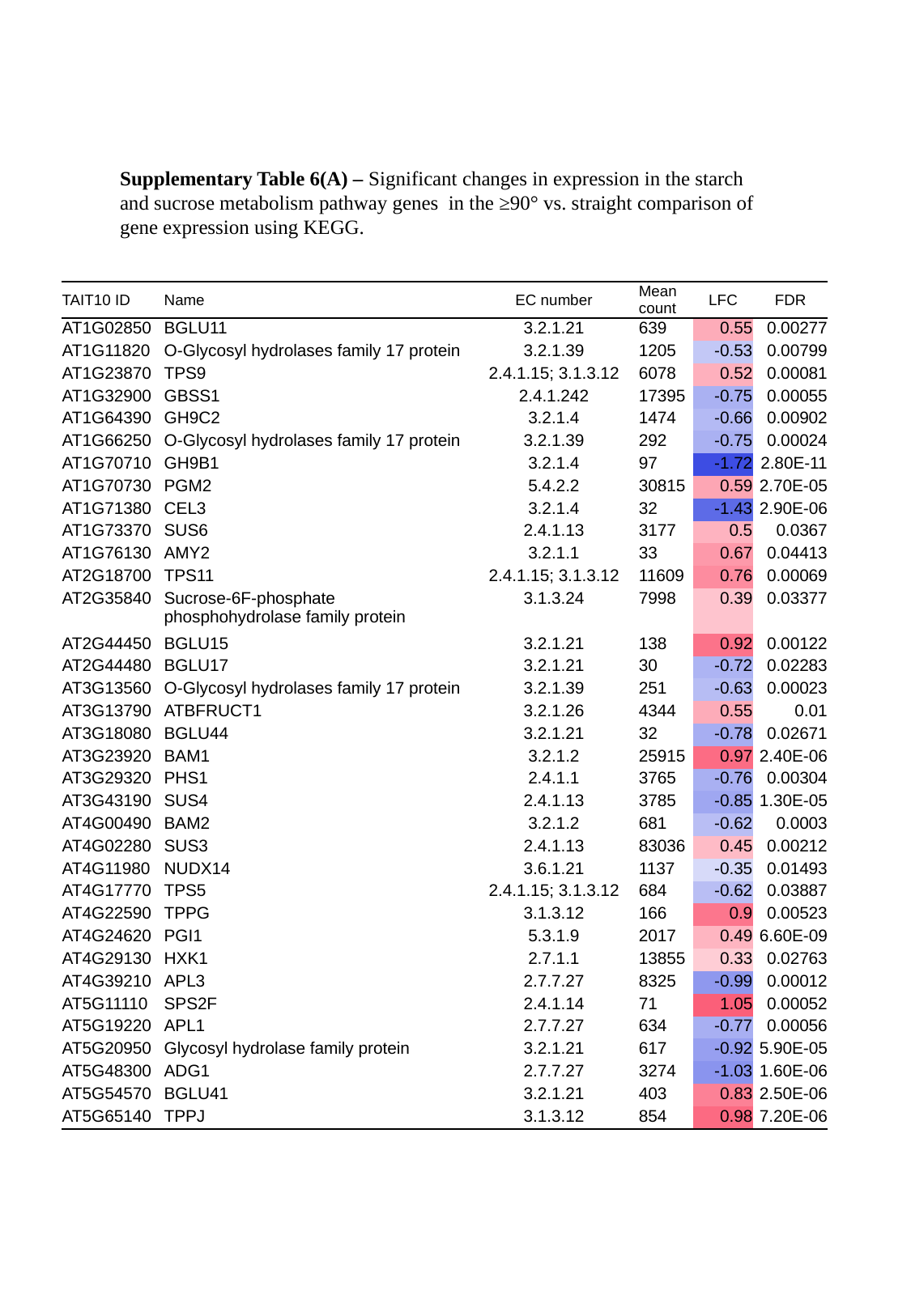

Supplementary Table 6(A) – Significant changes in expression in the starch and sucrose metabolism pathway genes in the ≥90° vs. straight comparison of gene expression using KEGG.
| TAIT10 ID | Name | EC number | Mean count | LFC | FDR |
| --- | --- | --- | --- | --- | --- |
| AT1G02850 | BGLU11 | 3.2.1.21 | 639 | 0.55 | 0.00277 |
| AT1G11820 | O-Glycosyl hydrolases family 17 protein | 3.2.1.39 | 1205 | -0.53 | 0.00799 |
| AT1G23870 | TPS9 | 2.4.1.15; 3.1.3.12 | 6078 | 0.52 | 0.00081 |
| AT1G32900 | GBSS1 | 2.4.1.242 | 17395 | -0.75 | 0.00055 |
| AT1G64390 | GH9C2 | 3.2.1.4 | 1474 | -0.66 | 0.00902 |
| AT1G66250 | O-Glycosyl hydrolases family 17 protein | 3.2.1.39 | 292 | -0.75 | 0.00024 |
| AT1G70710 | GH9B1 | 3.2.1.4 | 97 | -1.72 | 2.80E-11 |
| AT1G70730 | PGM2 | 5.4.2.2 | 30815 | 0.59 | 2.70E-05 |
| AT1G71380 | CEL3 | 3.2.1.4 | 32 | -1.43 | 2.90E-06 |
| AT1G73370 | SUS6 | 2.4.1.13 | 3177 | 0.5 | 0.0367 |
| AT1G76130 | AMY2 | 3.2.1.1 | 33 | 0.67 | 0.04413 |
| AT2G18700 | TPS11 | 2.4.1.15; 3.1.3.12 | 11609 | 0.76 | 0.00069 |
| AT2G35840 | Sucrose-6F-phosphate phosphohydrolase family protein | 3.1.3.24 | 7998 | 0.39 | 0.03377 |
| AT2G44450 | BGLU15 | 3.2.1.21 | 138 | 0.92 | 0.00122 |
| AT2G44480 | BGLU17 | 3.2.1.21 | 30 | -0.72 | 0.02283 |
| AT3G13560 | O-Glycosyl hydrolases family 17 protein | 3.2.1.39 | 251 | -0.63 | 0.00023 |
| AT3G13790 | ATBFRUCT1 | 3.2.1.26 | 4344 | 0.55 | 0.01 |
| AT3G18080 | BGLU44 | 3.2.1.21 | 32 | -0.78 | 0.02671 |
| AT3G23920 | BAM1 | 3.2.1.2 | 25915 | 0.97 | 2.40E-06 |
| AT3G29320 | PHS1 | 2.4.1.1 | 3765 | -0.76 | 0.00304 |
| AT3G43190 | SUS4 | 2.4.1.13 | 3785 | -0.85 | 1.30E-05 |
| AT4G00490 | BAM2 | 3.2.1.2 | 681 | -0.62 | 0.0003 |
| AT4G02280 | SUS3 | 2.4.1.13 | 83036 | 0.45 | 0.00212 |
| AT4G11980 | NUDX14 | 3.6.1.21 | 1137 | -0.35 | 0.01493 |
| AT4G17770 | TPS5 | 2.4.1.15; 3.1.3.12 | 684 | -0.62 | 0.03887 |
| AT4G22590 | TPPG | 3.1.3.12 | 166 | 0.9 | 0.00523 |
| AT4G24620 | PGI1 | 5.3.1.9 | 2017 | 0.49 | 6.60E-09 |
| AT4G29130 | HXK1 | 2.7.1.1 | 13855 | 0.33 | 0.02763 |
| AT4G39210 | APL3 | 2.7.7.27 | 8325 | -0.99 | 0.00012 |
| AT5G11110 | SPS2F | 2.4.1.14 | 71 | 1.05 | 0.00052 |
| AT5G19220 | APL1 | 2.7.7.27 | 634 | -0.77 | 0.00056 |
| AT5G20950 | Glycosyl hydrolase family protein | 3.2.1.21 | 617 | -0.92 | 5.90E-05 |
| AT5G48300 | ADG1 | 2.7.7.27 | 3274 | -1.03 | 1.60E-06 |
| AT5G54570 | BGLU41 | 3.2.1.21 | 403 | 0.83 | 2.50E-06 |
| AT5G65140 | TPPJ | 3.1.3.12 | 854 | 0.98 | 7.20E-06 |

## Slide 7
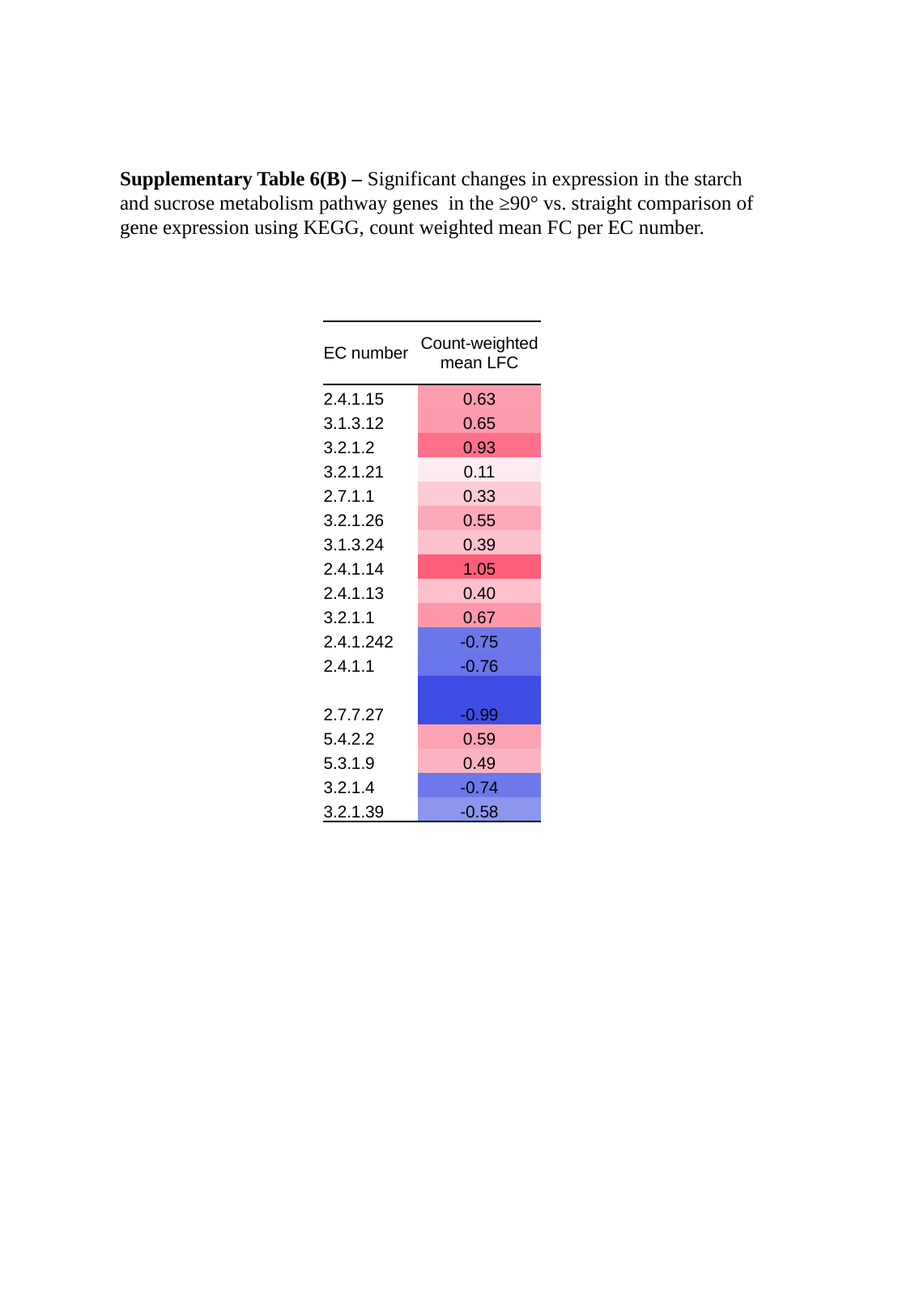

Supplementary Table 6(B) – Significant changes in expression in the starch and sucrose metabolism pathway genes in the ≥90° vs. straight comparison of gene expression using KEGG, count weighted mean FC per EC number.
| EC number | Count-weighted mean LFC |
| --- | --- |
| 2.4.1.15 | 0.63 |
| 3.1.3.12 | 0.65 |
| 3.2.1.2 | 0.93 |
| 3.2.1.21 | 0.11 |
| 2.7.1.1 | 0.33 |
| 3.2.1.26 | 0.55 |
| 3.1.3.24 | 0.39 |
| 2.4.1.14 | 1.05 |
| 2.4.1.13 | 0.40 |
| 3.2.1.1 | 0.67 |
| 2.4.1.242 | -0.75 |
| 2.4.1.1 | -0.76 |
| 2.7.7.27 | -0.99 |
| 5.4.2.2 | 0.59 |
| 5.3.1.9 | 0.49 |
| 3.2.1.4 | -0.74 |
| 3.2.1.39 | -0.58 |

## Slide 8
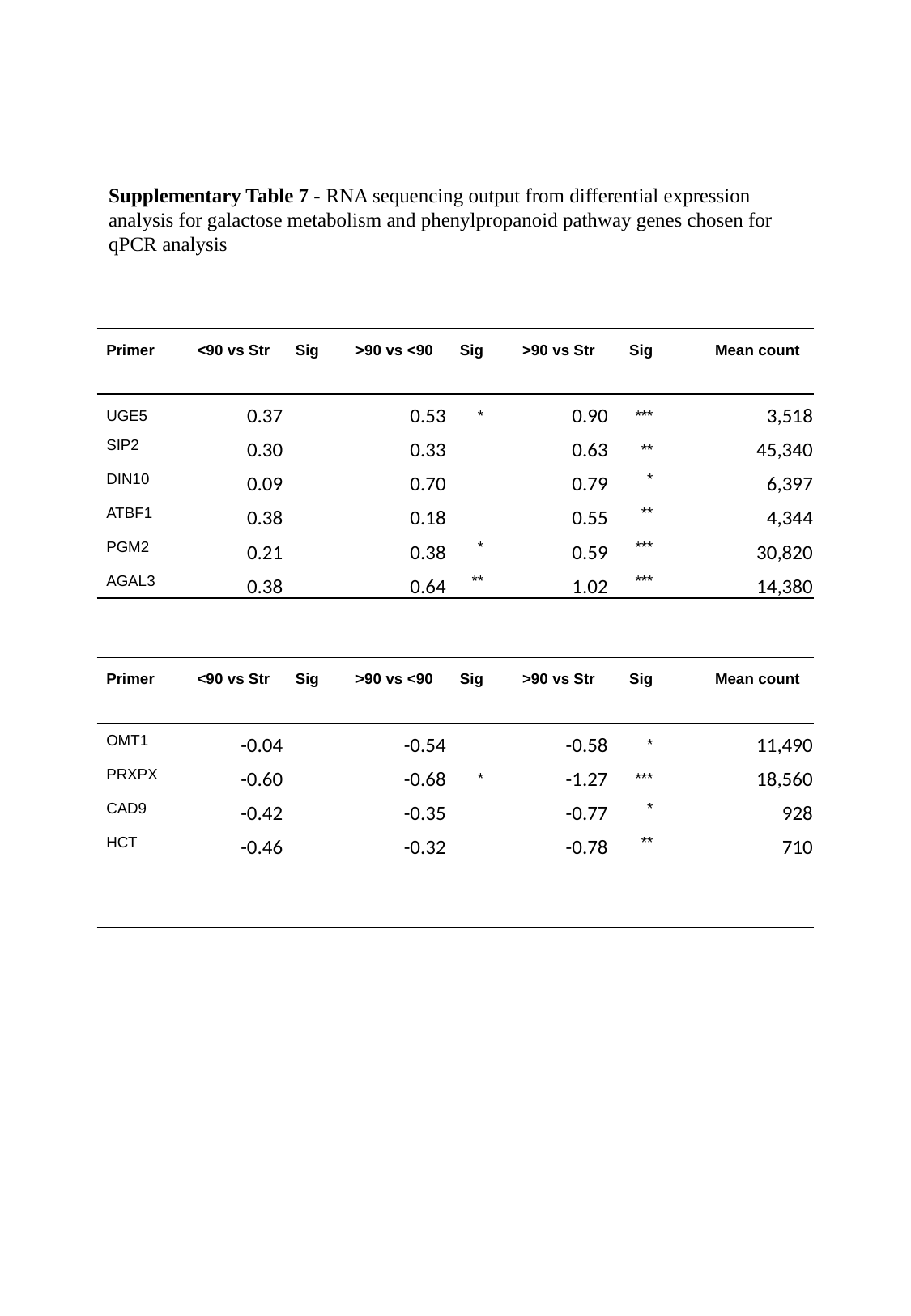

Supplementary Table 7 - RNA sequencing output from differential expression analysis for galactose metabolism and phenylpropanoid pathway genes chosen for qPCR analysis
| Primer | <90 vs Str | Sig | >90 vs <90 | Sig | >90 vs Str | Sig | Mean count |
| --- | --- | --- | --- | --- | --- | --- | --- |
| UGE5 | 0.37 | | 0.53 | \* | 0.90 | \*\*\* | 3,518 |
| SIP2 | 0.30 | | 0.33 | | 0.63 | \*\* | 45,340 |
| DIN10 | 0.09 | | 0.70 | | 0.79 | \* | 6,397 |
| ATBF1 | 0.38 | | 0.18 | | 0.55 | \*\* | 4,344 |
| PGM2 | 0.21 | | 0.38 | \* | 0.59 | \*\*\* | 30,820 |
| AGAL3 | 0.38 | | 0.64 | \*\* | 1.02 | \*\*\* | 14,380 |
| Primer | <90 vs Str | Sig | >90 vs <90 | Sig | >90 vs Str | Sig | Mean count |
| --- | --- | --- | --- | --- | --- | --- | --- |
| OMT1 | -0.04 | | -0.54 | | -0.58 | \* | 11,490 |
| PRXPX | -0.60 | | -0.68 | \* | -1.27 | \*\*\* | 18,560 |
| CAD9 | -0.42 | | -0.35 | | -0.77 | \* | 928 |
| HCT | -0.46 | | -0.32 | | -0.78 | \*\* | 710 |
| | | | | | | | |
| | | | | | | | |

## Slide 9
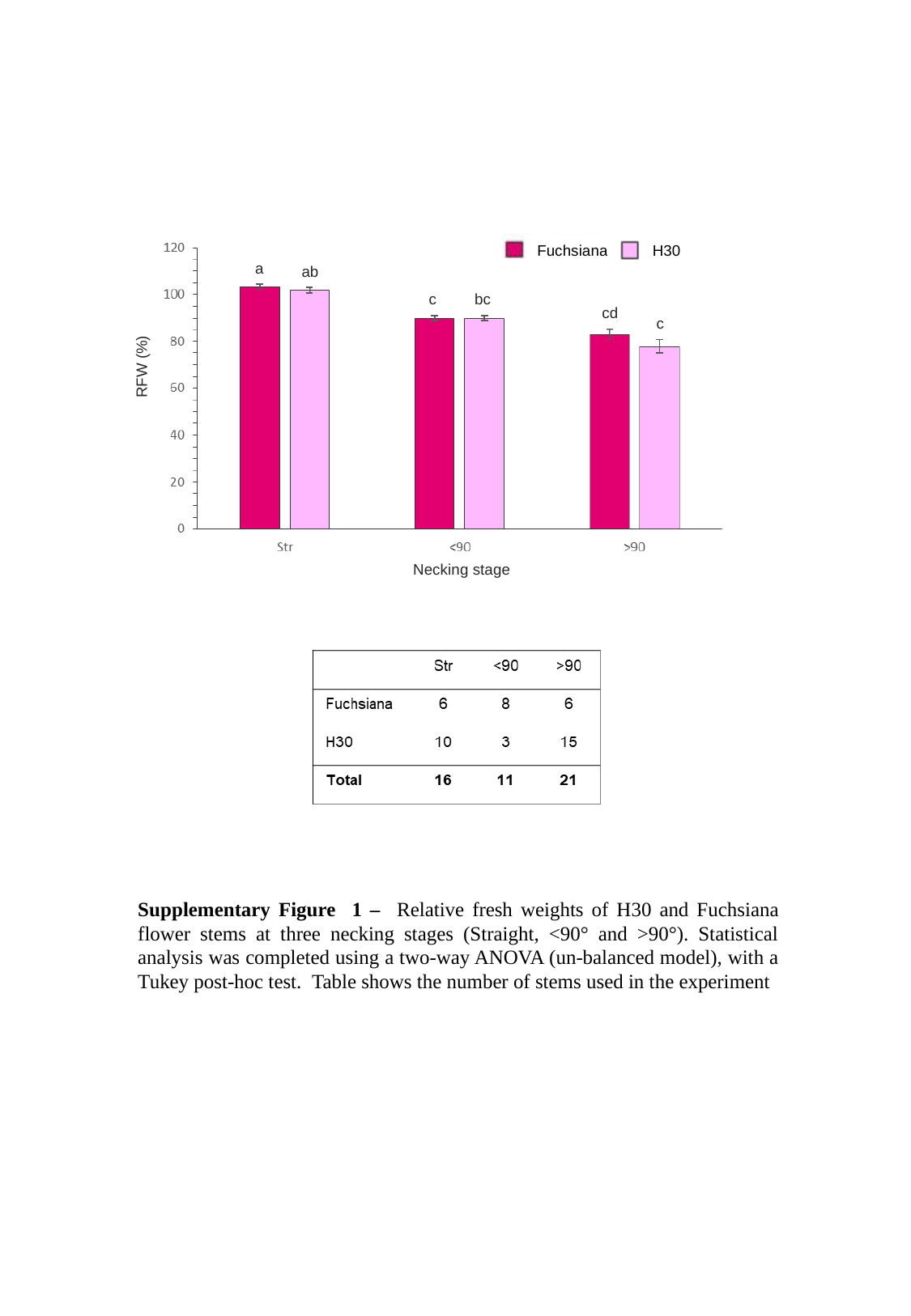

Fuchsiana
H30
a
ab
RFW (%)
c
bc
cd
c
Necking stage
Supplementary Figure 1 – Relative fresh weights of H30 and Fuchsiana flower stems at three necking stages (Straight, <90° and >90°). Statistical analysis was completed using a two-way ANOVA (un-balanced model), with a Tukey post-hoc test. Table shows the number of stems used in the experiment

## Slide 10
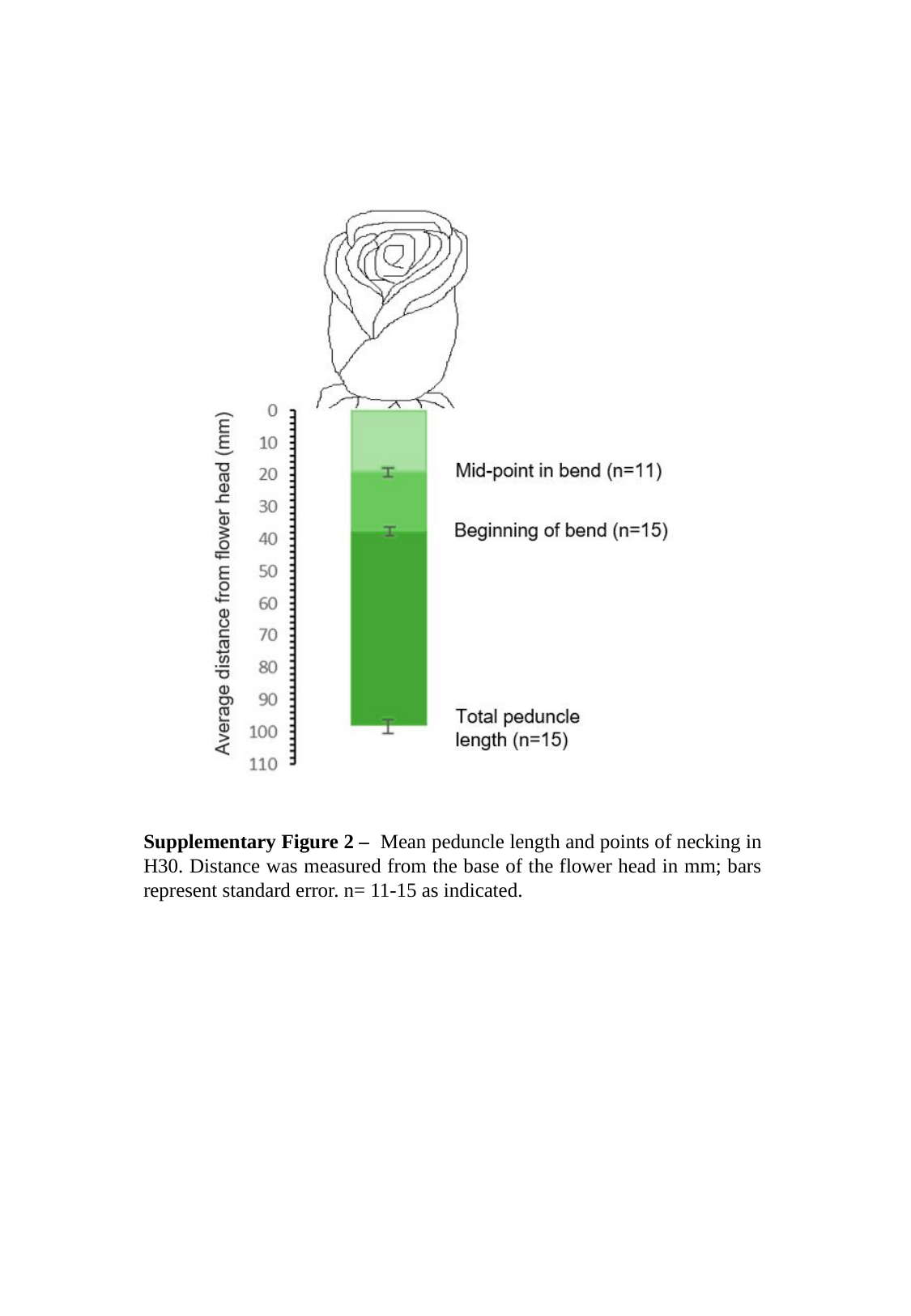

Supplementary Figure 2 – Mean peduncle length and points of necking in H30. Distance was measured from the base of the flower head in mm; bars represent standard error. n= 11-15 as indicated.

## Slide 11
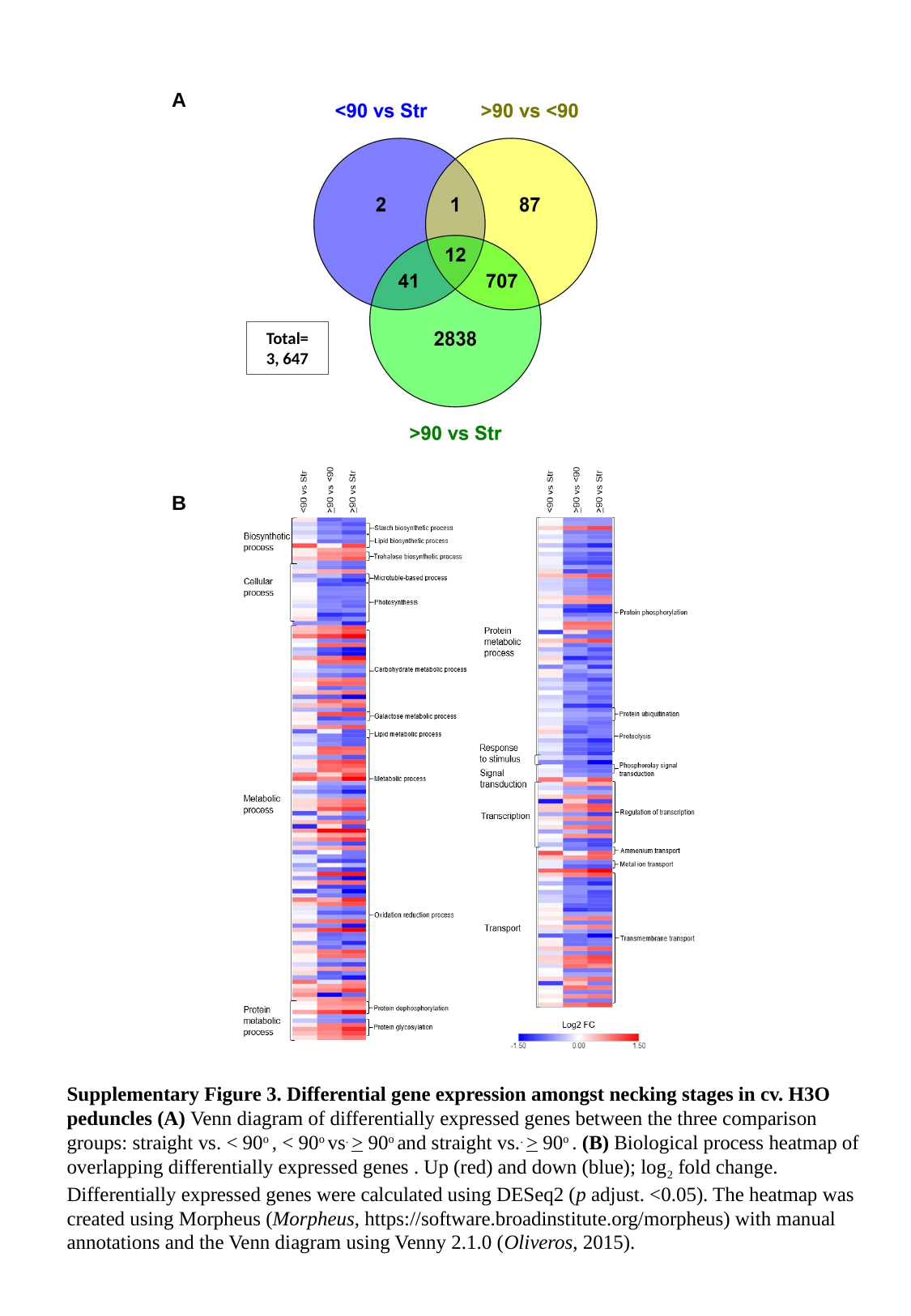

A
Total=
3, 647
B
Supplementary Figure 3. Differential gene expression amongst necking stages in cv. H3O peduncles (A) Venn diagram of differentially expressed genes between the three comparison groups: straight vs. < 90o , < 90o vs. > 90o and straight vs.. > 90o . (B) Biological process heatmap of overlapping differentially expressed genes . Up (red) and down (blue); log2 fold change. Differentially expressed genes were calculated using DESeq2 (p adjust. <0.05). The heatmap was created using Morpheus (Morpheus, https://software.broadinstitute.org/morpheus) with manual annotations and the Venn diagram using Venny 2.1.0 (Oliveros, 2015).

## Slide 12
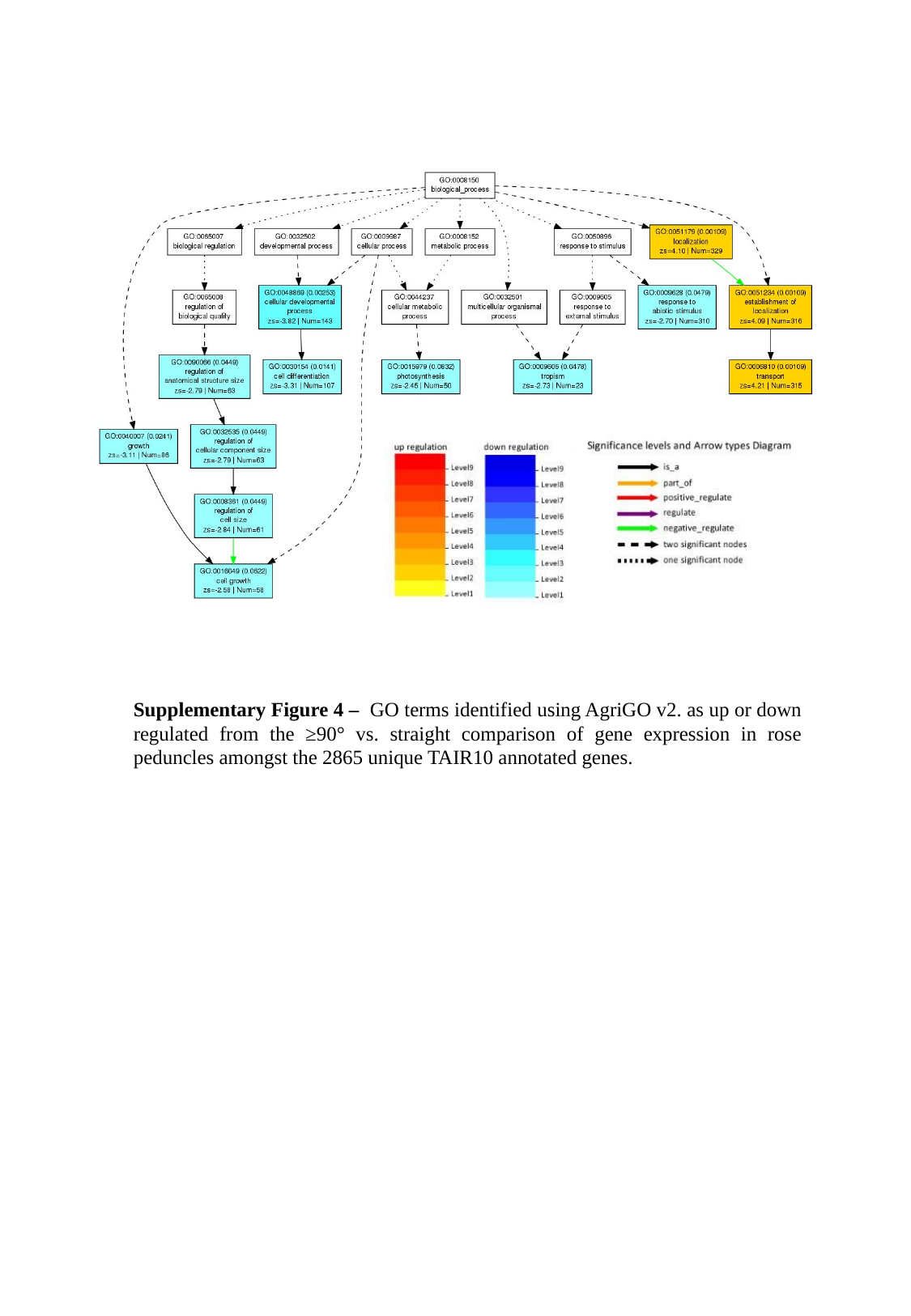

Supplementary Figure 4 – GO terms identified using AgriGO v2. as up or down regulated from the ≥90° vs. straight comparison of gene expression in rose peduncles amongst the 2865 unique TAIR10 annotated genes.

## Slide 13
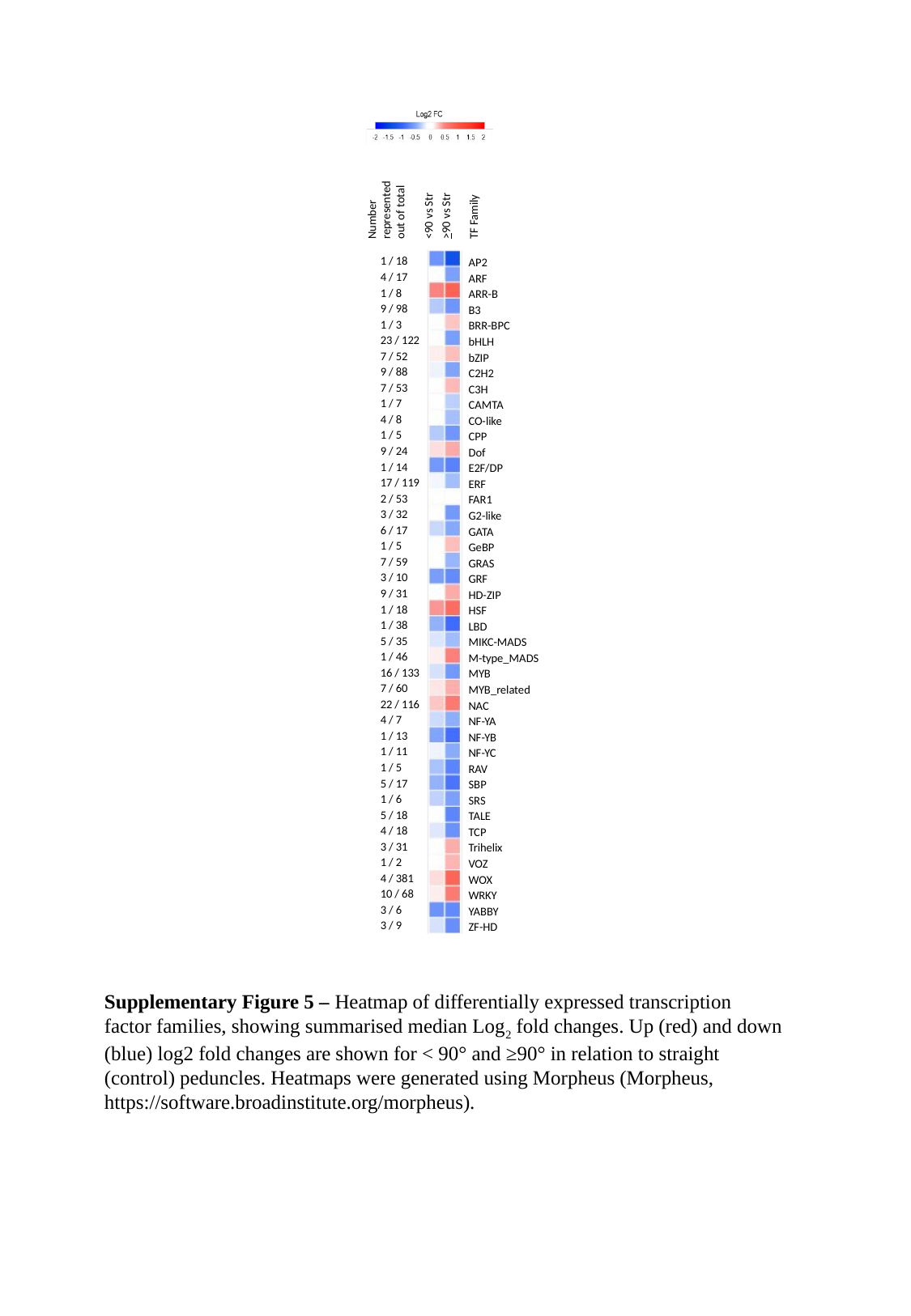

Number represented out of total
<90 vs Str
>90 vs Str
TF Family
1 / 18
4 / 17
1 / 8
9 / 98
1 / 3
23 / 122
7 / 52
9 / 88
7 / 53
1 / 7
4 / 8
1 / 5
9 / 24
1 / 14
17 / 119
2 / 53
3 / 32
6 / 17
1 / 5
7 / 59
3 / 10
9 / 31
1 / 18
1 / 38
5 / 35
1 / 46
16 / 133
7 / 60
22 / 116
4 / 7
1 / 13
1 / 11
1 / 5
5 / 17
1 / 6
5 / 18
4 / 18
3 / 31
1 / 2
4 / 381
10 / 68
3 / 6
3 / 9
AP2
ARF
ARR-B
B3
BRR-BPC
bHLH
bZIP
C2H2
C3H
CAMTA
CO-like
CPP
Dof
E2F/DP
ERF
FAR1
G2-like
GATA
GeBP
GRAS
GRF
HD-ZIP
HSF
LBD
MIKC-MADS
M-type_MADS
MYB
MYB_related
NAC
NF-YA
NF-YB
NF-YC
RAV
SBP
SRS
TALE
TCP
Trihelix
VOZ
WOX
WRKY
YABBY
ZF-HD
Supplementary Figure 5 – Heatmap of differentially expressed transcription factor families, showing summarised median Log2 fold changes. Up (red) and down (blue) log2 fold changes are shown for < 90° and ≥90° in relation to straight (control) peduncles. Heatmaps were generated using Morpheus (Morpheus, https://software.broadinstitute.org/morpheus).

## Slide 14
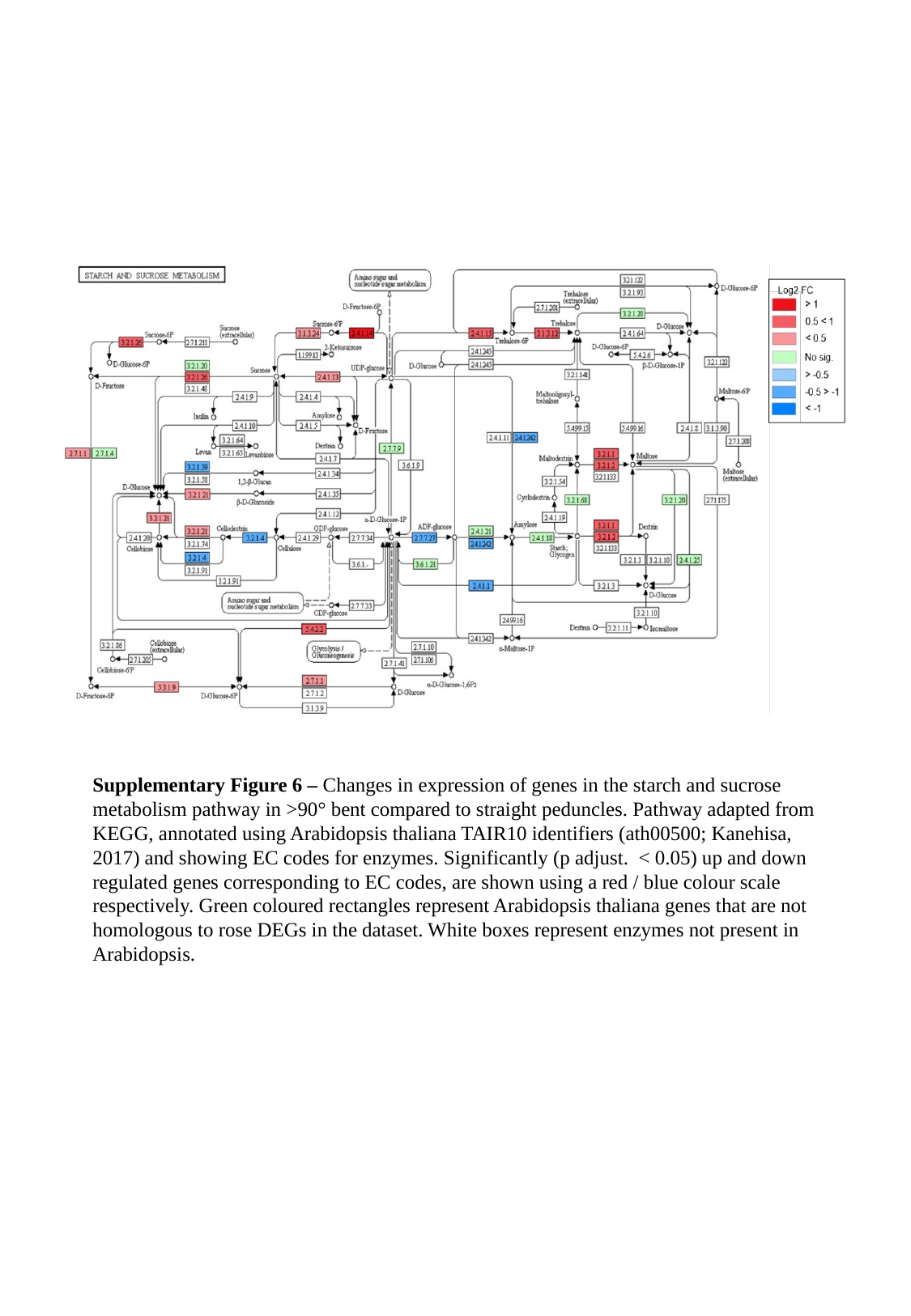

Supplementary Figure 6 – Changes in expression of genes in the starch and sucrose metabolism pathway in >90° bent compared to straight peduncles. Pathway adapted from KEGG, annotated using Arabidopsis thaliana TAIR10 identifiers (ath00500; Kanehisa, 2017) and showing EC codes for enzymes. Significantly (p adjust. < 0.05) up and down regulated genes corresponding to EC codes, are shown using a red / blue colour scale respectively. Green coloured rectangles represent Arabidopsis thaliana genes that are not homologous to rose DEGs in the dataset. White boxes represent enzymes not present in Arabidopsis.
